# Targeted Nanoparticle-Based Diagnostic and Treatment Options for Pancreatic Cancer

**DOI:** 10.3390/cancers16081589

**Published:** 2024-04-20

**Authors:** Xin Gu, Tamara Minko

**Affiliations:** 1 Department of Pharmaceutics, Ernest Mario School of Pharmacy, Rutgers, The State University of New Jersey, Piscataway, NJ 08554, USA; 2 Rutgers Cancer Institute of New Jersey, Rutgers, The State University of New Jersey, New Brunswick, NJ 08901, USA

**Keywords:** RNAs, CRISPR, immunotherapy, transferrin receptor (TfR), epidermal growth factor receptor (EGFR), somatostatin receptor 2 (SSTR2), vascular endothelial growth factor receptor (VEGFR), integrin, hyaluronic acid receptors, nanoparticle-based drugs

## Abstract

**Simple Summary:**

Pancreatic ductal adenocarcinoma (PDAC) accounts for 90% of pancreatic cancers. It is considered one of the deadliest cancers due to its high metastatic potential and drug resistance. This review discusses various targets and nanoparticle-based delivery systems developed, tested, and approved for the effective diagnosis and targeted treatment of PDAC.

**Abstract:**

Pancreatic ductal adenocarcinoma (PDAC), one of the deadliest cancers, presents significant challenges in diagnosis and treatment due to its aggressive, metastatic nature and lack of early detection methods. A key obstacle in PDAC treatment is the highly complex tumor environment characterized by dense stroma surrounding the tumor, which hinders effective drug delivery. Nanotechnology can offer innovative solutions to these challenges, particularly in creating novel drug delivery systems for existing anticancer drugs for PDAC, such as gemcitabine and paclitaxel. By using customization methods such as incorporating conjugated targeting ligands, tumor-penetrating peptides, and therapeutic nucleic acids, these nanoparticle-based systems enhance drug solubility, extend circulation time, improve tumor targeting, and control drug release, thereby minimizing side effects and toxicity in healthy tissues. Moreover, nanoparticles have also shown potential in precise diagnostic methods for PDAC. This literature review will delve into targeted mechanisms, pathways, and approaches in treating pancreatic cancer. Additional emphasis is placed on the study of nanoparticle-based delivery systems, with a brief mention of those in clinical trials. Overall, the overview illustrates the significant advances in nanomedicine, underscoring its role in transcending the constraints of conventional PDAC therapies and diagnostics.

## 1. Introduction

Pancreatic ductal adenocarcinoma (PDAC) represents 90% of all pancreatic malignancies and is known as one of the deadliest cancers, with a five-year survival rate of less than 5%, making it a persistent global health challenge [1]. It ranks as the fourth leading cause of cancer death in the United States, leading to over 30,000 deaths every year [2]. PDAC’s highly aggressive and metastatic nature, coupled with the lack of early detection methods, results in fewer than 20% of patients being diagnosed at localized and resectable stages, as 80–85% of advanced PDAC patients are ineligible for surgical intervention due to extensive metastasis [3]. Even those diagnosed in the early stages have a low postoperative five-year survival rate, merely around 20% [2,3,4,5].

The intricate nature of PDAC is marked by genetic heterogeneity, increased interstitial fluid pressure, and dense tumor stroma, creating a complex microenvironment [6,7,8]. This significantly enhances the metastatic potential and drug resistance, thereby obstructing the effective delivery of therapeutic agents by conventional chemotherapy treatment approaches. In response, research increasingly focuses on nanotechnology in therapy and diagnosis. As a revolutionary platform, nanoparticles can be highly multifunctional and demonstrate a high potential for treating various cancers [9,10,11,12,13,14,15,16]. They can be engineered to target markers on PDAC cells specifically, such as EGFR, VEGFR, transferrin, hyaluronan, integrins, and other molecules [17,18,19,20,21,22,23,24]. Additionally, nanocarriers can be conjugated with tumor-penetrating peptides like TAT, antennapedia, and iRGD to enhance intracellular penetration of delivered drug(s) [25,26]. This engineering enhances drug accumulation at tumor sites, modifies the tumor microenvironment, and can aid in immunotherapy and gene therapy. As a result, nanoparticles significantly enhance the therapeutic efficacy while minimizing damage to healthy tissues and reducing the side effects typically associated with conventional chemotherapy and radiation, making them a highly effective treatment option. Up to now, the FDA has approved two nanoformulations specifically for PDAC treatment, Onivyde and Abraxane [27]. Onivyde, a pegylated liposome formulation of irinotecan, combined with other chemotherapies, has shown promising outcomes in clinical practice compared to conventional gemcitabine-based therapies [28]. Abraxane, a nanoparticle albumin-bound formulation of paclitaxel, has been effective in combination with gemcitabine, offering better survival rates compared to gemcitabine alone [29]. These nanoparticle formulations represent a significant advancement in the targeted treatment of PDAC, offering new therapeutic approaches in this challenging field.

This comprehensive narrative literature review will examine the intricate microenvironment of PDAC and explore various diagnostic methods currently in use and under research, including nanotechnology-based techniques. It will then examine potential markers for targeted therapy, providing a detailed analysis of their roles and implications. Finally, we will discuss advanced nanoparticle delivery systems and their role in enhancing the efficacy of targeted therapies for PDAC treatment, highlighting recent breakthroughs and potential future directions in this area.

## 2. Epidemiology and Classification of Pancreatic Cancer

Although the primary determining factors of pancreatic cancer are not fully discovered, numerous studies suggest that smoking is a significant contributing factor [30,31,32]. Over the years, it has been reported that approximately 25% to 30% of pancreatic cancer cases can be attributed to smoking, and smokers are 2.5 to 3.6 times more likely to develop the disease. Furthermore, the risk of pancreatic cancer increases with extended exposure to tobacco [33].

In addition, family history is an established risk factor for pancreatic cancer. Research indicates that around 7% to 10% of pancreatic cancer patients have a family history of the disease [34]. For families with three or more first-degree affected individuals, the risk of pancreatic cancer is even higher, rising to 32 times compared to families without any history of the disease. In families with four or more PDAC individuals, the risk can escalate to 57 times higher [30,34]. Several case-control studies reveal that inherited factors such as obesity, diabetes mellitus, chronic pancreatitis, heavy alcohol consumption, familial breast cancer, and non-polyposis colorectal cancer may also heighten the risk of pancreatic cancer [30,32,34].

**Figure 1:**
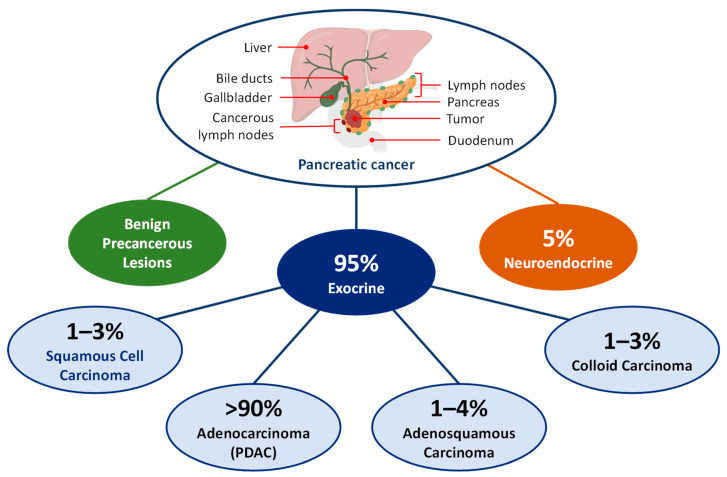
Classification of pancreatic cancer. PDAC—Pancreatic ductal adenocarcinoma.

Depending on their origin, pancreatic cancers are divided into two major types: exocrine and neuroendocrine (Figure 1) [31,32,35]. Exocrine glands, associated with the digestive system, secrete digestive enzymes. Exocrine pancreatic cancers, including various subtypes, make up 95% of pancreatic cancer cases. PDAC is the most common type of exocrine pancreatic cancer and accounts for more than 90% of cases. Squamous cell carcinoma, adenosquamous, and colloid carcinoma are rare exocrine pancreatic cancers. Cysts and other benign tumors forming in the pancreas can be precursors to pancreatic cancer.

Pancreatic carcinoma is a highly metastatic form of cancer that is difficult to detect in its early stages, leading to a poor prognosis [30]. Conversely, neuroendocrine gland cancers are less lethal and have a much better prognosis. These cancers affect islet cells that secrete hormones such as insulin and glucagon to regulate human blood sugar. Neuroendocrine carcinomas are relatively rare, accounting for less than 5% of all pancreatic cancers. This paper will focus on pancreatic ductal adenocarcinoma, which is the most fatal form of pancreatic cancer.

## 3. Diagnosis and Screening

### 3.1. Traditional Approaches

Early detection and diagnosis of pancreatic cancer remain challenging due to the lack of specific symptoms, biomarkers, and screening methods. Statistical analysis reveals a 10-year delay from the first emergence of the mutation to the development of nonmetastatic cancerous cells [36]. Because the pancreas is located behind the peritoneal cavity, pancreatic cancer often goes undetected in its early stages. Consequently, in most cases, common symptoms such as jaundice, abdominal pain, nausea/vomiting, and anorexia always indicate advanced cancer stages [31,32,37,38]. Unlike many other cancers, biomarkers currently do not play a pivotal role in the early detection of pancreatic cancer. Elevated carbohydrate antigen such as CA19-9 is a broadly studied and often-used screening tool in PDAC diagnosis and prognosis with a sensitivity of 79–81% and specificity of 82–90% in symptomatic patients, and 100% sensitivity and 98.5% specificity in asymptomatic patients. However, its positive predictive value (PPV) is only moderate at 72% in symptomatic patients and very low at 0.9% in asymptomatic patients, which makes it almost no clinical usage in practice for early detection [39,40]. Additionally, about 5–10% of Caucasians are Lewis antigen-negative, so they cannot produce detectable levels of CA19-9 [38,41]. Elevated CA19-9 can also occur in other cancers, such as colorectal and cholangiocarcinoma or in conditions like obstructive jaundice and liver cirrhosis, as well as in about 50% of PDAC tumors that are smaller than 3 cm, further complicating its use in clinical practice [42,43]. Although CA19-9 may not be an ideal option, it is currently the only FDA-approved marker for PDAC diagnosis, and it is reported to be the only most recommended tool in distinguishing PDAC and chronic pancreatitis, holding a sensitivity of 70–90% and a specificity of 68–91% [44]. Increased carcinoembryonic antigen (CEA) has also been reported in different cancers, including the pancreas, colon, lung, and breast [44]. Although reports show that around 30–60% of PDAC patients have elevated CEA [45], this biomarker has a dismal sensitivity of 25–54% and specificities of 75–91%, which is relatively low for practical use in clinics [46]. To enhance the accuracy and reliability of early diagnosis, many researchers are now focusing on combining CA19-9 and/or CEA with additional factors to create comprehensive panels of biomarkers [47,48,49,50].

Besides biomarker measurements, current PDAC diagnosis also relies on medical imaging. Conventional medical imaging techniques, including computed tomography (CT), magnetic resonance imaging MRI, endoscopic ultrasound (EUS), positron emission tomography (PET) scan, and X-ray angiography, have limited applicability in PDAC diagnosis due to their reduced accuracy. [37,38,51,52]. CT, in particular, is the primary modality for diagnosing and staging PDAC. A meta-analysis of 52 studies and 3567 patients suggested that CT-based investigation has a sensitivity, specificity, and diagnostic accuracy of 90%, 87%, and 89%, respectively [53]. However, this method remains a reliable diagnostic tool for tumors larger than 20 mm due to its relatively poor contrast resolution; for a smaller lesion, the sensitivity and accuracy decreased to 69% and 73%, respectively [52]. MRI shows superior sensitivity, specificity, and comparable diagnostic accuracy to CT, especially in small tumors and metastasis [52,53]. Nevertheless, its overall application is restricted by its cost and availability [52]. EUS, on the other hand, can particularly benefit patients with tumors smaller than 20 mm [51,52,54]. Pairing EUS with Fine Needle Aspiration (FNA) creates EUS-FNA, a technique that collects samples during an examination with minimal invasiveness [54]. This approach leads to an enhanced sensitivity of 92% and specificity of 96% [37,52,55]. Both medical imaging and biomarker evaluation necessitate highly skilled and experienced professionals, and inconsistent diagnosis may occur due to different physician training backgrounds [42]. Overall, more advanced techniques with uniform standards, high sensitivity, and specificity are critical to ensuring consistency and accuracy in PDAC diagnosis.

### 3.2. Advancement in Early Diagnosis and Screening

Given the limitation of accuracy and precision by conventional diagnostic and screening approaches, there is a pressing need for innovative detection methods targeting pancreatic cancer, especially in the initial stages. One promising technique is liquid biopsy, which permits minimal or non-invasive sampling to detect pancreatic cancer early or even precancerous lesions with high sensitivity [56]. Liquid biopsy can monitor tumor initiation, progression, and recurrence while providing real-time medication responses [37,57]. Researchers are investigating novel biomarkers from liquid biopsy samples collected via various routes, including saliva, pancreatic juice, bile, serum, feces, and urine. The biomarkers consist of circulating tumor cells, cell-free DNA, non-coding RNAs, and proteins, which can be combined with cutting-edge techniques like Clustered Regularly Interspaced Short Palindromic Repeats (CRISPR), nanotechnologies, and artificial intelligence (AI) [38,42]. These innovative methods complement the conventional methods, which may significantly improve the treatment outcomes in practice.

### 3.3. Extracellular Vesicles (EVs)

Extracellular vesicles (EVs) are exosomes, microvesicles, and apoptotic bodies released by cells into the extracellular space, enclosed by lipid bilayers containing cancer-related biomarkers such as proteins and nucleic acids [58]. Accurate removal of soluble contaminants like cells, small proteins, or other vesicles is crucial for the accurate isolation of EVs isolation and investigation of the biomarkers [58,59]. An advanced electrokinetic (ACE)-based platform called the Verita™ System has successfully purified EVs with high efficiency, enhancing the detection of pancreatic, ovarian, and bladder cancer with an average sensitivity of 71.2% at 99.5% specificity. This platform has facilitated the precise measurement of various protein biomarkers, including CA19-9. Notably, the detection of stage I pancreatic cancer by the innovative platform achieved 95.5%, demonstrating substantial promise in clinical application [59].

### 3.4. Proteomics

Proteomic biomarkers have been identified in various bodily fluids, including serum, urine, pancreatic juice, and bile. Serum and urine are preferred as collection methods due to their non-invasive nature. Prior studies revealed a significant correlation between cancer detection and serum proteins such as Glypican-1 (GPC1), Carboxypeptidase A4 (CPA4), C4b-binding protein α-chain (C4BPA), plasma free amino acids (PFAA), Mucin 5AC (MUC5AC), and serum osteopontin and tissue inhibitor of metalloproteinase 1 (OPNT + TIMP-1) [42]. Among them, the serum level of C4BPA is aberrantly higher in preoperative PDAC patients when compared to postoperative patients (*p* < 0.008 vs. *p* < 0.036). Importantly, C4BPA shows better potential in detecting stage I and II PDAC patients than CA19-9, with an ROC AUC of 0.912 vs. 0.737 [60]. Moreover, combined CA19-9 panels with other proteins such as C4BPA, MUC5AC, or OPNT + TIMP-1 often show much higher sensitivity in PDAC diagnosis [42,60]. Urine samples from early PDAC patients revealed elevated LYVE1, REGIA, and TFFI levels, demonstrating a higher AUC of 0.97 compared to 0.88 for CA19-9. Recent advancements utilizing iTRAQ-based analysis identified several diagnostic biomarkers [61,62,63]. Among them, the combination panel of PROZ, TNFRSF6B, and CA19-9 demonstrated a superior AUC of 0.932 for stage I PDAC [62]. Moreover, the combination of ALG-2 interacting protein X (ALIX) and CA19-9 has been reported to have an AUC of 0.91 compared to ALIX or CA19-9 alone, with a 90.6% sensitivity and an 83.9% specificity [61]. Buenafe et al. investigated cancer-related proteins LAMA5, SDCBP, and TENA in EVs from PDAC patients, indicating consistent upregulation of those EV proteins [57]. Novel biomarkers G protein-coupled receptor class C group 5 member C (GPRC5C) and epidermal growth factor receptor pathway substrate 8 (EPS8) were identified in detecting early-stage PDAC, and the combination panel demonstrated an AUC of 0.922 and 0.946, compared to the healthy individuals of the two groups, respectively [64]. The proteomic biomarkers mentioned above exhibit considerable potential for clinical application. However, their effectiveness still needs further validation and optimization, which are crucial to ensure their reliability and efficacy in early cancer detection.

### 3.5. Circulating Cell-Free DNA and Methylation

There is a marked increase in the concentration of circulating cell-free DNA (cfDNA) in the serum of patients with malignant lesions compared to healthy individuals and those with inflammatory conditions such as pancreatitis [38,65,66]. Although the biological cause for such an increase remains unclear, studies suggest that nucleic acids from cancer cell necrosis, apoptosis, or active secretion possibly cause it. cfDNA is predominantly released from hematopoietic cells, while various conditions, including pregnancy, organ transplantation, cancer, surgery, and radiation, can lead to increased cfDNA [67,68]. In cancer patients, cfDNA is originated from cancer cells and the tumor microenvironment, including non-malignant cells [69]. Circulating tumor DNA (ctDNA) is cfDNA released by circulating tumor cells (CTCs) originating from primary or metastatic tumors. Studies have shown that cfDNA contains genetic and epigenetic abnormalities in malignancies, including genomic mutations, copy number alterations, and hyper- and hypomethylation from CTCs or ctDNA [70,71]. Detecting and analyzing ctDNA can potentially identify cancer-related changes. Polymerase chain reaction (PCR) and Next-Generation Sequencing (NGS) are approaches used to detect cancer using ctDNA, and deep sequencing coverage, molecular barcoding, and error-suppression algorithms are utilized to enhance sensitivity and specificity [72]. In 1993, a study utilized allele-specific amplification through PCR to identify mutated K-ras in the plasma cfDNA of PDAC patients, which was later verified via direct sequencing. The altered codon matched the K-ras mutation in the tumor, indicating that ctDNA could potentially be used for PDAC diagnosis [73]. Subsequent studies have shown promising results with K-ras detection, particularly in the early phases [74]. The decrease of TP53 and KRAS mutations in cfDNA after treatment may predict PFS in PDAC patients [75]. Remarkably, even at stage I, when serum CA19-9 levels are normal, plasma K-ras mutation can be detected [76]. Sausen et al. identified 43% of K-ras mutations in stage II PDAC with a specificity of over 99.9% using digital polymerase chain reaction (dPCR) and NGS [77]. In 2019, Liu et al. developed a technique called single-strand library preparation and hybrid-capture-based circulating DNA sequencing (SLHC-seq), which enhances the sensitivity and accuracy of identifying somatic K-ras mutations in pre-cancerous intraductal papillary mucinous neoplasms (IPMNs) and early-stage PDAC, such as stage I and II, by restoring degraded and short ctDNA fragments [78]. These findings highlight the potential of ctDNA as a reliable PDAC diagnostic marker in clinical settings.

Epigenetic alterations are widely recognized as pivotal contributors to the initiation and progression of cancer, and DNA methylation can substantially alter tumor suppressor genes (via hypermethylation) and proto-oncogene (via hypomethylation) [79,80]. A study conducted in 2013 aimed to evaluate the methylation profiles of a set of genes closely associated with PDAC in serum samples from patients. It turned out that *ADAMTS1* and *BNC1* showed the highest mutation frequency of 92% and 68%, respectively. The combined panel exhibited a 75% sensitivity for detecting PanIN lesions and a 97% sensitivity for identifying stage I PDAC, which substantially outperformed the traditional CA19-9 biomarker, displaying only 20% and 52% sensitivities, respectively [81]. A follow-up study indicated that the two-gene panel possessed a sensitivity and specificity of 87.2% and 95.8%, as well as 64.1% and 93.7%, respectively, for detecting PDAC from healthy controls, PDAC, and pancreatitis patients. Impressively, this gene panel demonstrated a remarkable sensitivity of 97.4% and specificity of 91.6% for identifying all-stage PDAC cases, outperforming the traditional CA19-9 biomarker [82]. The researchers also added *LRFN5* and *PXDN* to the gene methylation panel of *ADAMTS1* and *BNC1*, which led to higher diagnostic accuracy (AUC 0.94). Furthermore, the investigators found that *LRFN5* and *PXDN* did not exhibit significant methylation frequency in patients with chronic pancreatitis (CP) compared to healthy individuals, providing better distinction than the panel of *ADAMTS1* and *BNC1* alone [83].

In a recent study, Shinjo et al. examined the methylation levels in five specific genes (*ADAMTS1*, *HOXA1*, *PCDH10*, *SEMA5A*, and *SPSB4*) in 47 cfDNA samples [84]. Using the methyl-CpG binding (MBD) protein coupled with a digital PCR method (MBD–ddPCR) technique, they found methylation in at least one of the five genes in 23 samples (49%). Previous studies proposed that K-ras mutation levels, rather than CA19-9, are more reliable for monitoring metastatic PDAC (mPDAC). However, post-therapy patients often show low methylation frequencies of RAS mutation and CA19-9, making them less ideal for tracking disease progression. In contrast, García-Ortiz et al. found that NPTX2 exhibited the highest methylation frequency (87.5%) in cfDNA of mPDAC patients and maintained a higher level of methylation after treatment, starting from a baseline of 100% methylation frequency [85]. Additionally, higher plasma NPTX2 methylation levels were strongly correlated with shorter survival periods in these patients. The findings indicate that monitoring methylation levels can be a promising tool to track the progression of mPDAC and its response to treatment.

### 3.6. Non-Coding RNA

Unlike protein-coding mRNA, non-coding RNAs do not encode any protein. They are primarily involved in regulating gene expression through epigenetic modification. During cancer development, these molecules exhibit altered expression patterns compared to healthy controls [86,87]. Previous studies demonstrated that specific types of non-coding RNAs, such as microRNAs (miRNAs), long non-coding RNAs (lncRNAs), and Circular RNAs (circRNAs), have great potential in accurately distinguishing early PDAC from healthy individuals.

#### 3.6.1. MicroRNAs (miRNAs)

miRNAs are small non-coding RNA molecules typically composed of 19-25 nucleotides, originating from primary microRNA (pri-miRNA). Mature miRNAs can bind to the 3′ UTR on a complementary sequence of the target mRNA transcript, thus regulating gene expression by repressing the translation or initiating the degradation of the target mRNA. Many PDAC-related miRNAs have been detected in body fluids like saliva, serum, and feces/urine specimens [88]. In fact, up to 81 markers have been identified in serum samples alone [89]. A substantial increase of approximately 12.1-fold in miRNA-21 and 11.6-fold in miRNA-155 expression was observed by RT-qPCR in non-invasive precursor lesions of PDAC compared to samples from healthy controls [90]. The findings demonstrate encouraging prospects for early medical interventions that may impede the progression of potential malignancies. Furthermore, compared to healthy controls and CP patients, PDAC patients exhibit a marked upregulation of four serum microRNAs, namely miRNA-21, miRNA-155, miRNA-210, and miRNA-196a. A combined panel of miRNA-16, miRNA-196a, and CA19-9 can distinguish between PDAC patients and CP/healthy groups with sensitivity, specificity, and accuracy of 87.7%, 97.7%, and 93.3%, respectively [91].

#### 3.6.2. Long Non-Coding RNAs (LncRNAs)

LncRNAs are RNA molecules that have more than 200 nucleotides. They have garnered increasing attention in recent years due to their critical regulatory roles in pancreatic tumorigenesis [92]. It was shown that lncRNA can influence DNA methylation by recruiting methyltransferase, such as DNMTs/TETs, to regulate tumor cell behavior, including proliferation, invasion, migration, apoptosis, autophagy, cell cycle, and promote resistance to radio- and chemotherapy [92]. A recent study identified immune-related lncRNAs, including LINC02325, FNDC1-AS1, ZEB2-AS1, and TEX26-AS1, significantly upregulated in PDAC tumor tissues [93]. Further research showed that reducing ZEB2-AS1 could increase E-cadherin expression meanwhile decreasing N-cadherin and Vimentin levels. The study suggests that ZEB2-AS1 may act as an oncogene by mediating the miR-204/HMGB1 axis and promoting tumor cell growth and migration by affecting the epithelial-mesenchymal transition (EMT) process in PDAC [94]. The diagnostic value of lncRNAs in PDAC has been evaluated and recognized by abundant studies. Long intergenic non-protein coding RNA (Linc-pint), a p53 transcriptional target, was observed to substantially decrease in plasma and tumor of PDAC patients, compared to healthy tissues, carcinoma of the ampulla of Vater (CAV) and cholangiocarcinoma (CCA), indicating its great potential in distinguishing PDAC from possible adjacent cancers. Combined with CA19-9, the ROC AUC improved from 0.78 (for CA19-9 alone) to 0.92 [95]. Upregulation of lncRNAs, such as salivary HOTAIR and PVT1, was found to be significantly associated with PDAC patients. The combined panel of the two genes accurately distinguished between PDAC patients and healthy individuals and differentiates the PDAC group from the benign pancreatic tumor (BPT) group with a sensitivity of 81.8% and specificity of 95.0% [96].

#### 3.6.3. Circular RNA (CircRNA)

CircRNA typically consists of 100 to 4000 base pairs in length. Instead of a linear shape formed by lncRNA and miRNA, it forms a covalently closed loop structure that links 5′ and 3′ ends [97]. This unique structure, which lacks free ends, enhances stability and resistance to degradation by RNase R, a 3′ to 5′ exoribonuclease. Additionally, circRNA is ubiquitously expressed in body fluids such as plasma, saliva, and urine, making it an excellent biomarker for cancer diagnosis [97]. A meta-analysis including six diagnostic studies suggested that circRNA could distinguish PDAC patients from healthy groups, with relatively high AUC, sensitivity, and specificity values of 0.86, 84%, and 80%, respectively [98]. Consequently, PDAC-related circRNAs holds great potential as a diagnostic tool for detecting precancerous pancreatic lesions and enabling earlier treatment.

#### 3.6.4. Clustered Regularly Interspaced Short Palindromic Repeats (CRISPR)

A novel diagnostic tool employing the CRISPR-Cas12a system has been developed to detect miRNA biomarkers with increased accuracy, specificity, and affordability in biological fluids. The EXTRA-CRISPR assay uses CRISPR-Cas12a to enable exponential and signal amplification of amplicons, replacing conventional linear rolling-circle amplification (RCA). It also features a modular padlock probe that simplifies the procedure and improves reaction kinetics. It integrates target-mediated ligation, RCA, Cas12a binding, and nucleolytic cleavage into complex reactions in one tube. The optimized assay can detect four PDAC biomarkers, miR-21, miR-196a, miR-451a, and miR-1246, in EVs isolated by commonly used methods. With a turnaround time ranging from 20 min to 3 h, this assay offers flexibility while providing results comparable to RT-qPCR without requiring a specialized instrument. Moreover, the assay maintains an impressive level of sensitivity with single-digit femtomolar concentration and single-nucleotide specificity [99]. The expedited workflow dramatically enhances the detection of miRNA biomarkers for diagnosing PDAC.

#### 3.6.5. Artificial Intelligence (AI)

As a rapidly developing technique, AI can be a powerful tool in helping with analyzing and processing data and images with high complexity, providing results with fewer biases and manual errors. The utilization of AI, comprised of machine learning (ML) and deep learning (DL), has excellent capability to assist in detecting PDAC at an early stage by analyzing medical images and biological markers [100].

### 3.7. Light-Based Pancreatic Cancer Diagnostic Techniques

A new light-based technique was proposed to identify precancerous and cancerous cysts in the pancreas [101,102]. This technique utilizes light-scattering spectroscopy to analyze subcellular structures within organs, providing diagnostic information without invasive procedures like tissue collection. By shining white light on cellular structures and analyzing the reflected and absorbed wavelengths, this method can distinguish between malignant and benign cysts in the pancreas. Unlike traditional methods that require tissue sampling, this so-called Virtual Biopsy Approach uses a tiny fiber optic probe connected to a broadband light source. By analyzing the reflected photons with an algorithm, real-time diagnostic results can be obtained without puncturing the cyst. This non-invasive technique could eliminate the need for painful and risky punctures and provide immediate results, which helps physicians quickly identify whether a cyst is cancerous or not without waiting for lab analyses. Clinical studies have reported the accuracy of this technique in identifying cysts to be as high as 95%.

The technique of light-scattering spectroscopy has the potential to enhance the early detection of pancreatic cancer, especially in cases involving cysts. This technology could revolutionize the diagnosis and management of pancreatic cancer in clinical settings by providing real-time diagnosis and replacing invasive procedures with non-invasive approaches. However, additional research and validation are required to refine this technique and make it more widely available for clinical use.

Laser Raman Spectroscopy (LRS) is a technique that uses light to differentiate between normal and malignant breast tissue. It is used to diagnose cancer during surgery. Precise and real-time analysis of Raman spectra can be achieved using statistical and machine-learning strategies. LRS is increasingly used in oncogenic diagnostics, but most algorithms fail to provide the two critical pieces of information the surgeon requires: the probability that the tissue classification is correct and the expected error in that probability. Stochastic backpropagation artificial neural networks trained using human experts and a classification algorithm can provide this information. This approach can help identify the additional contextual data needed to improve network classification performance and increase the correctness of the diagnosis.

### 3.8. Pancreatic Cancer and Microbiome Interaction

The microbiome, specifically the gut microbiome, has been increasingly recognized for its potential role in the pathogenesis of pancreatic cancer. Studies have indicated that alterations in the microbiota composition, known as dysbiosis, may influence the development, progression, and prognosis of Pancreatic cancer. The Human Microbiome Project has shed light on the impact of microbial communities on human health and disease, including cancer [103]. Advanced biological technologies such as high-throughput sequencing, transcriptomics, and metabolomics have enabled researchers to gain a deeper understanding of the role of the microbiome in pancreatic cancer [104,105]. Studies have shown that the interplay between the gut, urinary, and intra-pancreatic microbiome signatures can lead to local and systemic inflammation, immune responses, and the progression of pancreatic cancer. Certain microbial species and their metabolites have been shown to promote pancreatic cancer through mechanisms such as genotoxin-mediated mutagenesis or by fostering tumor-promoting inflammation while impairing immune surveillance [106].

### 3.9. Nanoparticles as Molecular Imaging Agents

The utilization of nanoparticles also holds promise for enhancing the precision of early PDAC detection. A biodegradable fluorescent polyplex nanoparticle has demonstrated a remarkable ability to specifically target the cholecystokinin-B receptor (CCK-BR), which is aberrantly expressed in PanIN lesions [107]. These PanIN lesions are often invisible by conventional imaging methods such as MRI, PET, or CT scans. The NP is constructed by attaching polyethylene glycol (PEG) with gastrin-10 peptide (Ga-10) for CCK-BR targeting and poly-L-lysine, linked to fluorescent Alexa Fluor 647 and 488. The effectiveness of the targeted nanoparticles was assessed in both wild-type mice and KC mice with the LSL-KrasG12D/+; P48-Cre (KC) genotype. Imaging outcomes and selective immunohistochemistry revealed a marked increase in fluorescence from targeted NPs at precancerous lesions in KC mice, especially in groups with advanced PanINs. Importantly, this enhanced fluorescence was not observed in other organs. This finding suggests that the biodegradable fluorescent polyplex NP could be a promising tool for early detection and treatment of PDAC.

The introduction of nanoparticles into the plasma of patients readily binds biomolecules, including proteins, lipids, sugar moieties, nucleic acids, and metabolites, creating a complex entity known as a personalized biomolecular corona (BC). An innovative approach for detecting early PDAC using blood samples, which involves characterizing BC formation through protein binding to Graphene Oxide nanoflakes was proposed [108]. These nanoflakes exhibited low binding affinity to abundant proteins like albumin while keeping strong adsorption to plasma proteins at low concentrations. In this study, Biomolecular Corona–Graphene Oxide components were analyzed via one-dimensional gel electrophoresis and categorized into distinct groups based on their molecular weights. Notably, the results showed that individuals with PDAC exhibited major bands within the 20–30 kDa range, whereas healthy volunteers displayed these bands within the 45–80 kDa range, demonstrating an ROC AUC of 0.96 and an elevated sensitivity of 92% [108]. Overall, these findings highlight the potential of nanotechnology-based approaches for early detection and treatment of PDAC.

### 3.10. Benefits and Drawbacks of the Pancreatic Cancer Diagnostic Approaches

Computed tomography scans are a type of medical imaging that can provide detailed cross-sectional images of the body. They are helpful in diagnosing pancreatic cancer because they can show the pancreas clearly. CT scans can also help determine if the cancer has spread to other nearby organs, lymph nodes, or distant organs, which is important for staging the cancer. Additionally, CT scans can be used to determine if surgery is a possible treatment option for pancreatic cancer. However, it is important to keep in mind that CT scans involve exposure to ionizing radiation, which can be a risk, especially with repeated scans. Some patients may also be allergic to the contrast dye used in CT scans, which can lead to potential adverse reactions. Finally, it is worth noting that CT scans may have limitations in differentiating between certain types of soft tissues within the body.

Magnetic resonance imaging scans utilize non-ionizing radiation, deemed safer than the ionizing radiation employed in CT scans. An MRI can produce detailed images of the pancreas and the surrounding structures, which can help diagnose and stage pancreatic cancer. It is particularly effective for individuals at high risk of pancreatic cancer or when searching for more minor metastatic spots in the liver. However, MRI machines may not be as widely available as CT scanners, which may cause delays in imaging. MRI scans usually take longer than CT scans, which can be challenging for some patients. Patients with specific metal implants or devices may be unable to undergo an MRI scan due to interference issues.

Endoscopic Ultrasound is a medical procedure more accurate than abdominal ultrasound in diagnosing and staging pancreatic cancer. It offers detailed imaging without significant surgery and allows doctors to obtain tumor biopsy samples during the procedure, aiding in definitive diagnosis. However, EUS still involves passing an endoscope through the digestive tract, which carries some risks. The accuracy of EUS may depend on the skill and experience of the endoscopist performing the procedure. Although rare, complications such as perforation or infection are possible with EUS procedures.

New techniques for diagnosing pancreatic ductal adenocarcinoma have the potential to significantly enhance the accuracy of traditional detection methods and reduce some possible negative effects. However, before these techniques can be widely used in clinical practice, further development and testing is needed. The use of nanotechnology can greatly improve the accuracy of diagnostics and prevent some of the limitations of conventional methods. Although they are being developed and tested, their applications are still in the early stages. 

## 4. Treatment Options for Pancreatic Cancer

**Figure 2:**
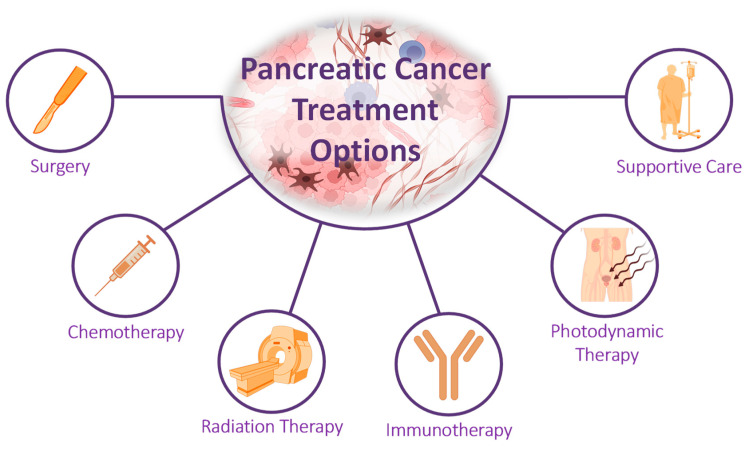
Treatment Options for Pancreatic Cancer.

The treatment of pancreatic cancer usually involves a combination of surgery, chemotherapy, radiation therapy, and other supportive measures (Figure 2). The goal of treatment is to remove the cancer, alleviate symptoms, and prolong survival.

Surgery is the primary treatment for pancreatic cancer when the tumor is localized and has not spread extensively. The most common surgical procedure is the Whipple procedure, which involves the removal of the head of the pancreas, the gallbladder, part of the small intestine, and nearby lymph nodes [109]. 

Chemotherapy uses drugs to kill cancer cells or stop them from growing. It is often used in combination with radiation therapy and surgery to increase the effectiveness of treatment. Chemotherapy can be administered intravenously or orally and may be given before or after surgery. Targeted therapies focus on specific characteristics of cancer cells, such as proteins or enzymes, to inhibit their growth and spread. This treatment option is often used in advanced stages of pancreatic cancer or when other treatments have failed [110].

Radiation therapy uses high-energy rays to destroy cancer cells. It can be used alone or in combination with surgery and chemotherapy. Radiation therapy can be delivered externally, where a machine targets the cancer cells from outside the body, or internally, where radioactive material is placed directly into the tumor [111,112]. In addition, laser therapy, specifically femtosecond laser irradiation, shows promise as a potential treatment for cancer and other diseases [113]. The effects of femtosecond laser irradiation were investigated on cancer cells using the T47D cell line as an in vitro model [114]. To conduct the study, cells were exposed to femtosecond laser irradiation at various wavelengths (UV, visible, and IR) at a constant power of 100 mW. Cell viability was measured directly and 24 h after femtosecond laser irradiation using an MTT assay. The results showed that femtosecond laser irradiation significantly inhibited breast cancer cell growth directly or 24 h after femtosecond laser exposure. Furthermore, the 420 and 440 nm wavelengths also demonstrated significant effects on cell viability. Notably, the 380 and 400 nm wavelengths, which were particularly effective, reassured us about the precision of the treatment. It was also observed that increasing exposure time enhanced the observed effect, with 10 min of exposure time being the most effective. However, the 700, 720, 750, and 780 nm wavelengths did not significantly affect cell viability with different exposure times. In conclusion, our study suggests that femtosecond laser irradiation could be a highly precise and effective treatment option for managing cancer.

Immunotherapy helps the immune system recognize and attack cancer cells. It is a relatively new treatment option for pancreatic cancer and has shown promise in some cases. However, its efficacy in pancreatic cancer is highly limited by the peculiar features of the pancreatic tumor microenvironment compared to other malignancies [115]. Consequently, a combination of immunotherapy and targeted therapy designed to suppress the resistance of tumor microenvironment may be a fascinating treatment approach. 

Photodynamic therapy (PDT) has emerged as a promising treatment option for pancreatic ductal adenocarcinoma or pancreatic cancer. The therapy involves using a photosensitizing agent, activated by light of a specific wavelength, to destroy targeted tissue selectively. In the case of PDAC, PDT has shown the potential to produce local necrosis in pancreatic tumors with acceptable morbidity. This minimally invasive treatment modality holds promise for effective treatment of pancreatic cancer, offering hope for improved outcomes and quality of life for patients suffering from this challenging disease. However, the effectiveness of PDT is limited by factors such as poor tumor selectivity, limited light penetration depth, and oxygen dependence. To overcome these challenges, researchers are exploring various strategies like finding new photosensitizers with higher photodynamic conversion efficiency, designing tumor-targeted PS, and using PDT-based combination therapies. These approaches aim to improve the overall efficiency of PDT for solid tumor treatment [116]. 

Supportive care involves managing symptoms, side effects, and the patient’s overall well-being. Such care can include pain management, nutrition, and psychological support [117].

It should be stressed that it is difficult to make future decisions about which patients with adjuvant agents (patients with extracellular vesicles) should receive. Most tumor boards do not consider stroma density when choosing adjuvant agents. The authors suggest addressing this issue by using artificial intelligence. Multiple modalities, including whole genomic sequencing, radiomic, and pathomic analysis, can be used to achieve personalized medicine.

Genomic integration with pathomic analysis is a technique that combines genetic information with detailed pathology data for a better understanding of diseases at the molecular level. This approach enables healthcare providers to develop more precise diagnosis, prognosis, and treatment strategies tailored to individual patients. Genomic data provides information about an individual’s genetic makeup, including variations in genes that may influence disease susceptibility, progression, and response to treatment. By analyzing genomic data, healthcare providers can identify specific genetic markers associated with certain diseases or drug responses, enabling personalized treatment plans based on an individual’s unique genetic profile. Pathomic analysis involves the study of tissue samples at a microscopic level to characterize disease processes, such as cellular morphology, protein expression patterns, and tissue architecture. Integrating genomic data with pathomic analysis allows for a deeper understanding of how genetic alterations manifest at the tissue level and contribute to disease development and progression. The integration of genomic and pathomic analysis provides an accurate and early diagnosis of various diseases by identifying specific genetic mutations and pathological changes related to different conditions. By merging genomic and pathomic data, healthcare providers can develop personalized treatment plans that target the underlying molecular mechanisms of a disease, resulting in more effective therapies with fewer side effects. The combination of genomic and pathomic information offers valuable insights that can predict disease outcomes based on both genetic factors and tissue characteristics, allowing for better patient management and monitoring. 

Recently, we proposed an individual approach for treating ovarian carcinoma, which can be successfully used for treating other cancers, including PDAC [118]. This approach includes the analysis of the expression of genes responsible for the development and progression of the selected cancer type and cancer cell resistance to chemotherapy. As the result of the measurement in tumor samples, the expression of genes/proteins involved in these processes, a mixture of nanoparticle-based tumor-targeted delivery systems containing siRNAs targeted to these proteins is selected and used for cancer chemotherapy. The results of pre-clinical in vivo evaluation of the approach showed the efficacy of the individual personalized treatment (selection of drugs/siRNAs based on the individual tumor genetic profile) when compared with traditional chemotherapy (one drug fits all) or precision (selection of drugs based on average characteristics of the population) treatment.

In the past decade, radiomics has been the focus of many studies that showcase its potential through retrospective proof-of-concept research [119]. However, most of these studies employed non-replicable and heterogeneous methods, resulting in varied outcomes. For radiomics to prove its clinical impact, it has to now shift towards open-science and independent databases and adopt standardized practices as recommended by the Image Biomarker Standardization Initiative. Additionally, researchers must explore innovative research paths incorporating other ‘-omics’ data to better understand the relationships between imaging of STS, gene-expression profiles, and tumor microenvironment. Recently, the computational pathology research community has shifted its focus from replicating pathologists’ diagnostic processes to discovering and unlocking “sub-visual” prognostic image cues from histopathological images [119,120]. As our knowledge and experience in digital pathology grow, the emerging goal is to integrate other omics or modalities to create a better prognostic assay. Some authors have proposed using artificial intelligence, specifically multiple modalities, including whole genomic sequencing, radiomic, and pathomic analysis, as a path toward personalized medicine. In recent years, machine learning and pathomics pipelines have improved cancer diagnostics and prognostics for entities like breast and prostate cancer. The initial step in these pipelines is to identify and segment the tumor area, usually performed automatically to save time. Therefore, a multi-task convolutional neural network that can balance disease detection and segmentation accuracy was proposed [121].

In this manuscript, we will focus on discussing potentially effective treatment mechanisms of PDAC, particularly emphasizing using targeted drug delivery systems for these purposes.

## 5. Targeted Mechanisms and Pathways

**Figure 3:**
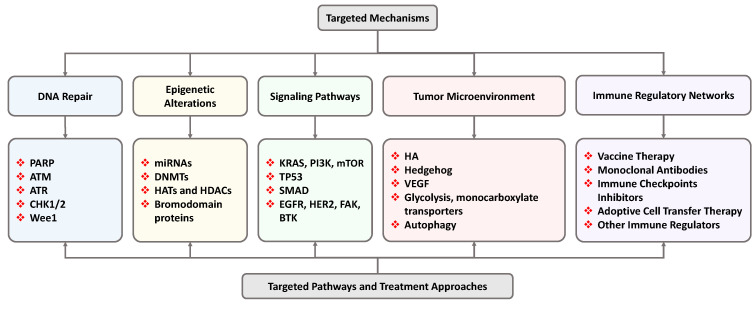
Targeted mechanisms, pathways, and approaches in the treatment of pancreatic cancer.

Analysis of completed, ongoing, and planned clinical trials devoted to treating PDAC allowed us to select the most promising therapeutic targets, mechanisms, and approaches for effective chemotherapy of PDAC [110]. The primary targeted mechanisms, pathways, and approaches are briefly summarized in Figure 3, created based on the data presented in [110]. The main goal of chemotherapy is to induce cancer cell death with a toxic agent and, if possible, simultaneously suppress mechanisms that are responsible for the efflux of a toxic substance and repair damages caused by the drug as well as its detoxification. Previously, we termed these two primary mechanisms of cancer resistance to chemotherapy as pump and nonpump resistance, respectively [122,123,124,125,126,127,128,129,130]. Although the suppression of pump and nonpump resistance in cancer cells alone may, to some extent, induce cancer cell death, it should be however stressed that the maximum chemotherapy efficacy can be achieved only by the simultaneous induction of cell death and suppression of resistance mechanisms. Such a two-pronged attack can rarely be achieved by one cytotoxic agent, which requires a multifunctional and multicomponent system that includes a cell death inducer and one or more suppressors of cancer cell resistance. The nanotechnology approach provides an effective way of achieving such a multi-pronged task by including several active ingredients in one or several complex systems [123]. It should also be stressed that such complex multifunctional anticancer systems possess extremely high cytotoxicity. Therefore, only a local or targeted delivery of such biologically active agents, specifically to pancreatic cancer cells, can significantly enhance the treatment efficacy and limit adverse side effects on healthy cells and tissues. Below, we will briefly discuss possible therapeutic targets and treatment approaches to suppress cell resistance to chemotherapy in PDAC based on the literature data [110,131,132,133,134].

### 5.1. Targeting DNA Repairing

DNA damage response pathways are critical in maintaining genomic stability and repairing DNA damage caused by internal or external impacts. Because the main mechanism of cell death induction by many chemotherapeutic agents is DNA damage, repair DNA damages caused by a chemotherapeutic toxic agent represents the major player in nonpump resistance of many types of cancers, including PDAC. Consequently, the suppression of such processes represents a potentially effective approach to enhancing the efficacy of the treatment of PDAC. Because the primary mechanism of cell death induction by many chemotherapeutic agents is DNA damage, repair of DNA damages caused by a chemotherapeutic toxic agent represents the major player in nonpump resistance of many types of cancers, including PDAC. Consequently, suppressing such processes represents a potentially practical approach to enhancing the efficacy of the treatment of PDAC. The inhibition of following targets is being explored to suppress DNA repair mechanisms in PDAC: PARP—Poly (ADP-ribose) polymerase; ATM—Ataxia Telangiectasia Mutated; ATR—ataxia telangiectasia and Rad3-related protein; DNA-PK—DNA-dependent protein kinase; CHK1/2—Checkpoint kinase ½, Wee1—the Scottish dialect word wee, meaning small—a nuclear kinase belonging to the Ser/Thr family of protein kinases.

### 5.2. Targeting Epigenetic Alterations

**Figure 4:**
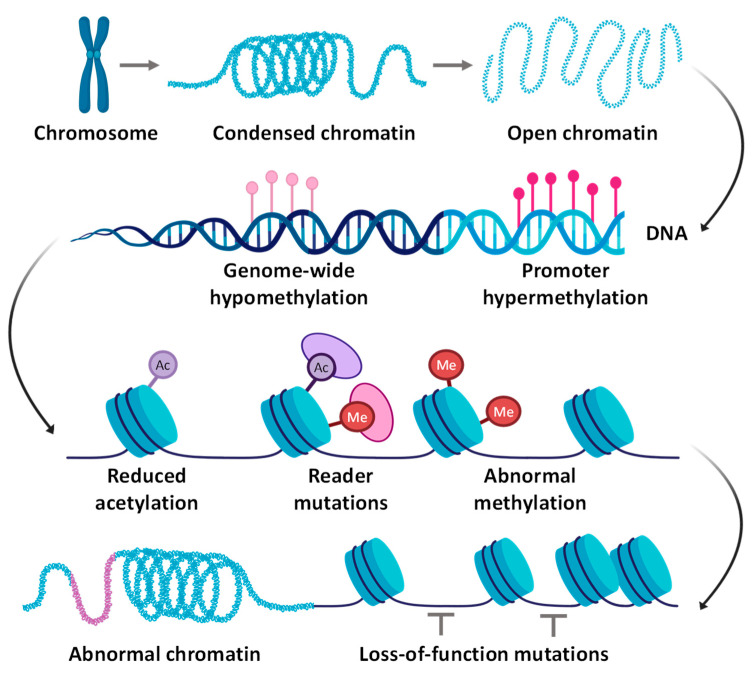
Mechanisms of cancer epigenetics (redrawn from [141]).

Epigenetic alterations refer to gene expression or function changes that do not involve modifications to the DNA sequence itself [135]. Epigenetic alterations play a crucial role in the pathogenesis of pancreatic cancer. These changes on the DNA level include DNA hyper and hypomethylation, reduced acetylation, histone modifications, and loss-of-function mutations in non-coding DNA, leading to abnormal chromatin structure (Figure 4). Such alterations can also include non-coding RNA regulation. Aberrant epigenetic modifications can lead to the overexpression or silencing of tumor suppressor genes and oncogenes, resulting in uncontrolled cell growth, invasion, and metastasis [136,137,138,139,140].

Several types of biological molecules involved in epigenetic alterations have been explored as potential targets for the treatment of pancreatic cancer, including miRNAs (MicroRNA), DNMTs (DNA methyltransferases), HATs, and HDACs (histone deacetylase and histone acetyltransferases), bromodomain proteins. MicroRNAs are non-coding RNAs interacting with mRNA, leading to degradation or reduced translation [142]. miRNAs regulate and are regulated by several vital pathways that involve cell differentiation, proliferation, and apoptosis [143]. DNA methyltransferase 1 (DNMT1) is required for DNA methylation during replication [144]. It was found that pancreatic cancer stem cells demonstrated hypermethylation via DNMT1 upregulation, and the suppression of DNMT1 in pancreatic stem cells reduced their self-renewal and in vivo tumorigenic potential [145]. Inhibition of histone deacetylase and histone acetyltransferases leads to increased or decreased histone acetylation, respectively, reactivates tumor suppressor gene expression, suppresses proliferation in cancer cells, and induces apoptosis [146]. The bromodomain (BRD) and extra terminal domain (BET) families of proteins recognize acetylated lysine residues and regulate molecular interactions in transcriptional control. Suppressing some members of this protein family also led to the death of PDAC cells [147]. In summary, affecting epigenetic alterations represents a promising strategy for treating PDAC. However, their application required additional investigation and an individual approach with stratification of patients by the expression of the mentioned proteins alone with the limitation of severe adverse side effects of their inhibitors.

### 5.3. Targeting Key Signaling Pathways

Numerous inhibitors of signaling pathways have been tested for cancer treatment [110]. Their main targets include the following protein families: (1) KRAS, PI3K, mTOR—Kirsten rat sarcoma virus, Phosphoinositide 3-kinases, mammalian target of rapamycin; (2) TP53—tumor protein 53; (3) SMAD4—The abbreviation refers to the homologies to the Caenorhabditis elegans SMA (“small” worm phenotype) and MAD family (“Mothers Against Decapentaplegic”) of genes in Drosophila; (4) EGFR, HER2, FAK, BTK—epidermal growth factor receptor, human epidermal growth factor receptor 2, focal adhesion kinase, Bruton’s tyrosine kinase, proteins involved in tyrosine kinase signaling pathways. These signaling pathways are governed or closely involved in regulating proliferation, survival, and metastases in many cancer cells, including PDAC. However, such key signaling pathways are active in normal cells. Consequently, systemic delivery of drugs and biologicals targeted to these pathways can potentially induce severe side effects upon healthy organs, tissues, and cells. The application of nanotechnology approaches that allow for targeted delivery of active substances directly to the PDAC may augment their anticancer efficacy and limit the adverse side effects.

### 5.4. Targeting the Tumor Microenvironment and Related Metabolic Reprogramming

One of the key factors contributing to the poor prognosis of pancreatic cancer is its complex tumor microenvironment (TME). The TME plays a crucial role in the development and progression of pancreatic cancer by providing a protective shield for tumor cells, supporting angiogenesis, and promoting metastasis. This complex environment consists of various cell types, including cancer-associated fibroblasts, immune cells, endothelial cells, and tumor-associated macrophages. These cells interact with tumor cells and the extracellular matrix, leading to the reprogramming of tumor cell metabolism and the formation of a supportive niche that facilitates tumor growth and progression. Consequently, targeting the TME as well as metabolic reprogramming has emerged as a promising strategy for improving the efficacy of pancreatic cancer treatment [148,149,150,151]. 

Due to their specificity, biocompatibility, and ease of production, nanoscale-targeted drug carriers are a promising solution for preventing immune escape and improving the antitumor immune response at various phases of tumor growth and inhibition pathways in the TME [152,153]. Through a tissue microarray analysis, it was discovered that there were significant differences in the number of bacteria found in tumors versus normal tissues. To target these bacteria, researchers developed mesoporous silica nanoparticles decorated with bacterial lipoteichoic acid (LTA) antibodies (LTA-MSNs), which could be used to deliver antitumor drugs [154]. The LTA-MSNs were able to target bacteria in tumors precisely. In mice with different types of cancer, the intravenous administration of the bacteria-targeted nanoparticles demonstrated a high tumor-targeting ability. This bacteria-guided tumor-targeting strategy has excellent potential for differential drug delivery and cancer treatment. 

On the other hand, using live bacteria that target tumors provides a unique and effective therapeutic option for cancer treatment [155]. These bacteria have versatile capabilities for suppressing cancer, which makes them stand out from other therapies. They accumulate and proliferate within tumors, where they can initiate antitumor immune responses. Moreover, they can be programmed via genetic manipulation or synthetic bioengineering to produce and deliver anticancer agents based on clinical needs. This therapeutic approach using live tumor-targeting bacteria can be applied as a monotherapy or combined with other anticancer therapies to achieve better clinical outcomes.

### 5.5. Targeting Immune Regulatory Networks

The tumor microenvironment in pancreatic cancer is a complex environment that is characterized by the presence of various immunosuppressive cell populations. These cells play a crucial role in cancer cells’ immune evasion and resistance to therapy. Some critical immunosuppressive cell populations in pancreatic cancer and their main functions are presented below [156].

#### 5.5.1. Tumor-Associated Macrophages (TAMs)

TAMs are derived from monocytes that infiltrate the tumor and differentiate into macrophages. Cytokines and growth factors produced by these cells contribute to tumor development, angiogenesis, and metastasis.

#### 5.5.2. Myeloid-Derived Suppressor Cells (MDSCs) 

MDSCs are a heterogeneous population of immature myeloid cells that suppress adaptive and innate immune responses. Their accumulation in TME produces immunosuppressive cytokines and reactive oxygen species that inhibit the activation and function of T cells.

#### 5.5.3. Neutrophils

Neutrophils are the most abundant white blood cells in the human body and are known to have both pro- and antitumor effects. In pancreatic cancer, neutrophils, however, exhibit an immunosuppressive phenotype that promotes angiogenesis and metastasis.

#### 5.5.4. Regulatory T Cells (Tregs)

Tregs are a subset of T cells that suppress immune responses and maintain self-tolerance. As a result of the accumulation of Tregs in the TME of pancreatic cancer, immune evasion is promoted by suppressing the activation and function of effector T cells.

Various strategies have been developed to target immunosuppressive cell populations in pancreatic cancer and enhance the immune response against tumors [157,158]. Some of these strategies include [115,158,159]:

- Immunotherapy: Immunotherapy involves using agents that stimulate or modulate the immune system to recognize and attack cancer cells. By blocking the inhibitory signals cancer cells use to evade the immune system, checkpoint inhibitors, such as anti-PD-1 and anti-CTLA-4 antibodies, have shown promise in treating advanced pancreatic cancers.
- Combination Therapies: Combining immunotherapy with chemotherapy, radiation therapy, or targeted therapies enhances the immune response against tumors and improves patient outcomes.
- Targeting Immunosuppressive Cell Populations: Strategies for inhibiting or depleting immune suppressive cell populations, such as TAMs, MDSCs, neutrophils, and Tregs, have been developed and verified in clinical trials.
- Immunomodulatory Agents: The use of immunomodulatory agents, such as cytokines and costimulatory molecules, is being explored as a potential treatment for pancreatic cancer to enhance the immune system’s antitumor activity.

Overall, the TME of pancreatic cancer is characterized by a highly immunosuppressive environment that promotes immune evasion and resistance to therapy. Understanding the complex interplay between cancer cells and immunosuppressive cell populations is crucial for developing effective treatment strategies. The use of immunotherapy, combination therapies, targeting immunosuppressive cell populations, and immunomodulatory agents holds promise for improving patient outcomes in advanced pancreatic cancer. The intricate microenvironment, invasive characteristics, and immunosuppressive nature of PDAC tumors make it very challenging for effective drug delivery. Standard treatments only offer modest improvements in overall survival and patient quality of life. Targeted treatment strategies could potentially improve the delivery of medications to tumor sites and lead to better therapeutic outcomes. Nanotechnology has the potential to help deliver these treatment agents specifically to pancreatic cancer cells to enhance the efficacy of treatment and limit adverse side effects on healthy organs, tissues, and cells.

## 6. Nanotechnology Approaches for Treating Pancreatic Cancer

Nanoparticles have gained significant attention in medicine due to their potential for delivering therapeutic agents. Nanoparticles for drug delivery offer several advantages, including enhanced drug solubility, improved drug stability, targeted delivery, and reduced systemic toxicity. One of the main advantages of using nanoparticles for drug delivery is their ability to improve the solubility of poorly soluble drugs. Many therapeutic agents, particularly in cancer treatment, have limited solubility in water, which can hinder their effectiveness when administered through traditional methods. Nanoparticles can encapsulate these drugs, increasing their solubility and bioavailability, thereby improving their therapeutic efficacy. Furthermore, nanoparticles can enhance the stability of therapeutic agents. Some drugs are susceptible to degradation or inactivation when exposed to physiological conditions or enzymatic activity. Encapsulating these drugs within nanoparticles can improve their stability, leading to a longer shelf life and better preservation of their pharmacological activity. In addition, nanoparticles enable targeted drug delivery to specific sites within the body. Through surface modifications and functionalization, nanoparticles can be designed to selectively accumulate in diseased tissues or cells while minimizing exposure to healthy tissues. This targeted approach improves the therapeutic outcome and reduces the potential for off-target effects and systemic toxicity. Moreover, using nanoparticles allows for the controlled release of therapeutic agents over an extended period. By engineering the properties of nanoparticles, such as size, shape, and composition, drug release kinetics can be tailored to achieve sustained and controlled release profiles. This controlled release mechanism can optimize drug concentrations at the target site and minimize the frequency of administration. 

It should be stressed that the application of nanoparticle-based drug delivery for treating pancreatic cancer has attracted considerable attention and research efforts in recent years. More than a thousand papers on this topic have been published in recent years [160]. The main nanotherapeutic approaches focus on nanoparticle-based delivery systems that improve the effectiveness of PC immunotherapy [161,162] and the application of various drug delivery systems for improving pharmacokinetic properties and anticancer efficacy of known and novel drugs [163]. However, despite a considerable number of publications on this field of research, only limited types of nanoparticle-based drugs have reached clinical trials with limited therapeutic success. Nevertheless, in our honest opinion, only the application of tumor-targeted multifunctional drug delivery systems has the potential to overcome challenges in the effective treatment of PDAC and to prevent adverse side effects on healthy organs, tissues, and cells. Targeting nanoparticles to pancreatic cancer cells can also avoid their entrapment by macrophages and prevent immunological and inflammatory processes associated with phagocytosis [164]. Consequently, here we will discuss how different types of nanoparticles can be used for the delivery of therapeutic drugs and nucleic acids with a specific focus on targeting mechanisms and potential plasma membrane receptors and other molecules which ligands can potentially serve as targeting moieties to deliver drugs specifically to the PDAC cells in order to utilize described above mechanisms.

### 6.1. Nanoparticles

**Figure 5:**
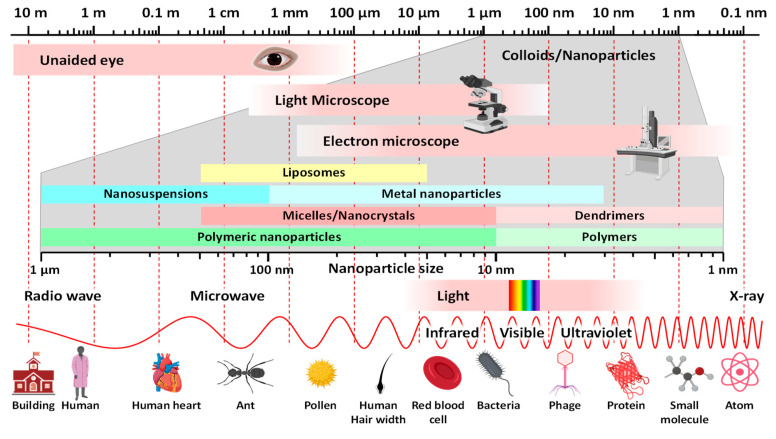
Nanoparticles. Reproduced from [15].

**Figure 6:**
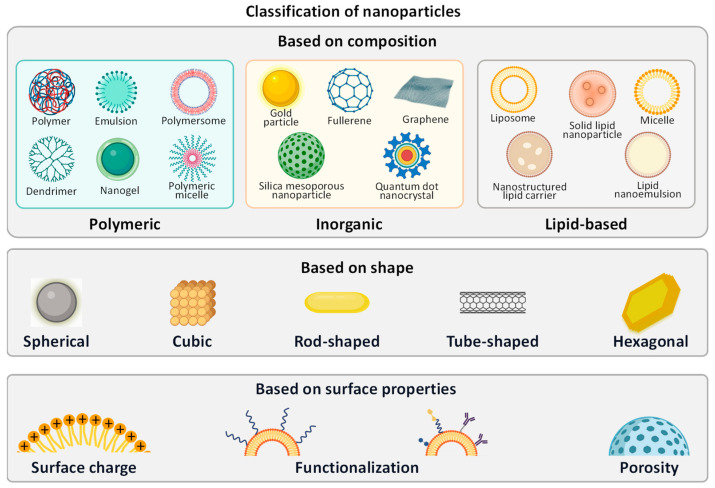
Classification of nanoparticles based on different criteria.

By definition, colloidal particles that fall within the range of 1 nm to 1000 nm in size are nanoparticles (Figure 5) [15]. They cannot be seen by the naked eye, cannot be separated by filtration, and have a size comparable with the wavelength of visible and ultraviolet light, bacteria, phages, proteins, and small molecules. However, the lower limits of micro- and nanoparticles remain vague, and nanoparticles smaller than 500 nm are generally preferred for therapeutic purposes [15]. Over the past few decades, nanoparticles have been thoroughly investigated because of their enormous potential for improving drug delivery. Generations of nanoparticle drugs have been created for cancer therapy, beginning with Doxil (PEGylated Liposomal Doxorubicin). Developed by Janssen for ovarian cancer and Kaposi’s sarcoma, it was a breakthrough when the FDA approved it in 1995, leading to more advances in this area [165]. Nanoparticle-based drug delivery systems contain various platforms, including polymer nanoparticles, dendrimers, lipid-based nanoparticles, nanospheres, and magnetic nanoparticles, offering numerous advantages for diagnosis, imaging, and therapy (Figure 6). These highly versatile systems can be tailored for active targeting and functional modifications like tumor-penetrating, enhancing drug accumulation in target areas, and improving overall efficacy. Their higher drug loading capacity reduces the necessary dosing frequency, thereby minimizing toxicity and adverse effects. 

Classifying nanoparticles is essential for understanding their behavior, applications, and potential risks [166]. Nanoparticles can be classified based on various factors such as their composition, size, shape, and surface properties (Figure 6). It should be emphasized that the above classification is somewhat subjective, does not claim to be complete, and represents only one version. Nevertheless, a detailed understanding of nanoparticles’ structure and various modifications is critical for further discussion of the problem of use for therapeutic purposes and for the treatment of prostate cancer. Nanoparticles can be made using various materials. It is logical to categorize their chemical structure into three main classes: polymeric, inorganic, and lipid-based (Figure 6). They also can be made from any combination of these materials, forming composite structures. The internal structure of nanoparticles from each class can vary substantially from simple geometries like gel, metal, or lipid spheres to more complex designs like dendrimers and porous or nanostructured nanoparticles. As a result, such structures (or their combinations) can form nanoparticles of different overall shapes, including spheres, cubic structures, rods, tubes, and hexagonal or more complex shapes. In addition, the chemical composition of the materials making up the nanoparticles can be chosen to have a negative or positive surface charge. Such a charge can be constant or change its magnitude or sign depending on environmental conditions (temperature, pH, and several other factors). Charged nanomaterials can be used to form more complex structures (for example, for conjugation with nucleic acids for delivery of the latter) or directly to control the mechanisms of intracellular delivery, increase their toxicity, and several other applications [9,10,12,13,14,15]. In addition to surface charge, other modifications to the outer surface of nanoparticles can be used. In particular, modification of the surface of poly(ethylene glycol) (PEG) furnishes the so-called STEALTH properties by dramatically reducing the capture of microparticles by the endothelial system, protecting them from rapid destruction on the way to the final site and increasing the circulation time of these particles in the blood [165]. Several ligands can also be straightly attached to the nanoparticle’s surface or through a linker for active targeting and functional modifications like tumor-penetrating, enhancing drug accumulation in target areas, and improving overall efficacy [11,12,13,20,21,22,128,159,167]. Various carbohydrates, antibodies, and other molecules can be used to increase nanoparticle specificity. 

### 6.2. Lipid-Based Nanoparticles

#### 6.2.1. Liposomes

Liposomes are one of the most researched nanoparticles ever since their discovery by Alec Bangham in the 1960s [168]. Liposomes are spherical nanoparticle vesicles made of lipid bilayers formed by self-assembly of amphiphilic and biocompatible phospholipids such as soy PC (soy phosphatidylcholine), egg PC (egg phosphatidylcholine), DSPE (1,2-distearoylsn-glycero-3-phosphoethanolamine), HSPC (hydrogenated phosphatidylcholine from soybean lecithin), and DSPC (1,2-distearoyl-glycero-3-phosphocholine), among others [169,170]. While the hydrophobic tails group with one another, the hydrophilic head groups point toward the inner core and aqueous environment, encapsulating hydrophilic drugs within their internal core and lipophilic drugs between their lipid bilayers. Numerous excipients, including steroids, polymers, and membrane proteins, can change liposome permeability, stability, fluidity, and drug-release properties [171]. As mentioned above, they can also evade the RES through surface modifications like PEG conjugation, and active targeting can be accomplished by coupling ligands to cell surface receptors (Figure 6). Due to their biocompatibility, biodegradability, and versatility, liposomes have found numerous potential applications for drug delivery and gene delivery.

Onivyde, or pegylated liposomal irinotecan (topoisomerase I inhibitor), was approved by the FDA back in 2015 to be used as a second-line treatment for metastatic PDAC, specifically for stages after gemcitabine therapies, in conjunction with fluorouracil and leucovorin [28]. A feasibility study on patients with advanced solid tumors revealed that the concentration of SN-38, the active metabolite of irinotecan, is about five times higher in tumors than in plasma at 72 h (*p* = 0.013) [172]. A preclinical study comparing liposomal and unencapsulated forms of irinotecan found that the peak plasma levels of irinotecan were ten times higher with the liposomal form than with the unencapsulated form. However, in contrast, the peak levels of SN-38 were ten times lower when liposomal irinotecan was used. The results suggested that before being released from liposomes, a large amount of irinotecan can be held internally and prevented from converting into SN-38 by carboxylesterase (CES) enzymes. Furthermore, high intratumoral concentrations of irinotecan and SN-38 were observed after 168 h following the administration of liposomal form. In contrast, over 90% of irinotecan was cleared from tumors within 24 h after injection of free drug. According to the team’s definition, SN-38 duration refers to the amount of time that the concentration of SN-38 remains at or above 120 nmol/L to maintain its antitumor effects. Compared to unencapsulated irinotecan, liposomal irinotecan has a significantly longer tumor SN-38 duration of over 100 h than the 40 h of unencapsulated irinotecan. This prolonged duration leads to an extended exposure to tumor cells, resulting in enhanced antitumor activity and response to treatment [173]. In the following NAPOLI-1 phase III study, 417 gemcitabine refractory mPDAC patients were treated with liposomal irinotecan alone or in combination with fluorouracil plus folinic acid, as compared to treatment with only fluorouracil plus folinic acid. The combined therapy reached a median overall survival of 6.1 months, outcompeting the 4.2 months of fluorouracil plus folinic acid [174]. In 2023, a NAPOLI-3 phase III trial report investigated the therapeutic effects of NALIRIFOX (combination of liposomal irinotecan, oxaliplatin, leucovorin, and fluorouracil) vs. nab-paclitaxel plus gemcitabine. Notably, NALIRIFOX outperformed nab-paclitaxel plus gemcitabine, showing a median overall survival rate of 11.1 months as opposed to 9.2 months, reducing the risk of death by 27% (hazard ratio 0.83) [175]. A US-based institutional report found that patients who were given NALIRIFOX as their second-line treatment after gemcitabine-based therapy had a better outcome. Their median OS was 23 months, and their median PFS was 4.8 months, which was noticeably longer than the patients who received NALIRIFOX as their third-line or later treatment after receiving gemcitabine-based therapy, who had an OS of only 4.1 months and a PFS of only 2.2 months. The results indicated that using NALIRIFOX at earlier stages may significantly improve survival outcomes. Importantly, NALIRIFOX had lower grade 3–4 treatment-related hematological adverse effects like neutropenia, anemia, and peripheral neuropathy than nab-paclitaxel plus gemcitabine despite having more instances of GI disorders like diarrhea and vomiting.aken together, liposomal therapeutic approaches like Onivyde hold great potential as a primary treatment option for patients with a manageable safety profile.

#### 6.2.2. Solid Lipid Nanoparticles

Solid lipid nanoparticles (SLNs) are one of the most extensively studied lipid-based nanoparticles. SLNs are colloidal systems consisting of a hydrophobic solid core surrounded by a phospholipid monolayer dispersed in an aqueous surfactant solution or water [176]. SLNs were developed as an advanced colloidal system to overcome some limitations of conventional nanoparticles such as liposomes, (micro)emulsions, and polymeric nanoparticles, designed to prolong drug release, enhance cellular absorption, increase the availability of therapeutic compounds, decrease drug resistance, and ultimately boost the effectiveness of the therapy. A key advantage of SLNs is that their preparation does not require organic solvents, significantly reducing toxicity. Moreover, the improved synthesis process involving high-pressure homogenization is more cost-effective and effortless to scale up for more extensive production. Unlike liquid phase encapsulation in liposomes, solid core offers enhanced stability and superior release control by entrapping drugs inside. The hydrophobic nature provides better accommodation for drugs with poor aqueous solubility, which accounts for a significant part of newly developed therapeutic agents on the market. By exploiting different synthetic techniques and lipid materials, both hydrophilic and lipophilic drugs can be incorporated into SLNs to create intricate complexes such as homogeneous matrices of solid solutions, drug-enriched shells, or drug-enriched cores [176]. SLNs enable precise delivery of drugs and genes, allowing for specific and regulated release, which can benefit various applications. A recent study proposed a formulation encapsulating nimesulide, an NSAID and potential inhibitor of the KRAS/PTEN signaling pathway, in SLNs. This method aims to boost bioavailability, reduce hepatotoxicity, and inhibit cell growth in PanIN cells by increasing PTEN levels [177]. In order to improve the efficacy of the PDAC standard treatment drug gemcitabine, a 2019 study evaluated the impact of a gemcitabine-loaded SLN (Gem-SLN) on MiaPaCa-2 and patient-derived primary pancreatic cancer cell lines (PPCL-46). The study found that Gem-SLN demonstrated a significantly lower IC50 than gemcitabine hydrochloride (GemHCl) treatment in both cell lines. The improvement was especially notable in the PPCL-46 group, where the IC_50_ values were reduced by 4.67-fold in 2D and 3.65-fold in 3D cell culture models, demonstrating its superior anticancer activity [178]. SLN particles are also effective in reversing multidrug resistance (MDR) by using different cellular entry mechanisms than those employed by drug-sensitive cells. Paclitaxel-loaded SLNs (Ptx-SLN) were tested for their antitumor activity and cellular uptake in drug-sensitive MCF-7 and MDR MCF-7/ADR human breast cancer cell lines [179]. The results showed that Ptx-SLN significantly reduced the survival rate of chemotherapy-resistant cells compared to paclitaxel delivered in DMSO and Cremophor EL with ethanol. Further investigation into cellular uptake revealed that the intracellular accumulation of Ptx-SLN and Rhodamine-SLN decreased when genistein (Gen), a caveola-mediated endocytosis inhibitor, was used. However, promazine (Cpz), an inhibitor of clathrin-mediated endocytosis, did not change the uptake in MCF-7/ADR cells. Interestingly, no change in SLN uptake was observed in the MCF-7 cell group with either Gen or Cpz. These results suggest that SLN can exploit caveola-mediated endocytosis in MCF-7/ADR cells to reverse drug resistance and enhance drug delivery, ultimately improving antitumor efficacy. Cationic SLN can also form a lipoplex with negatively charged nucleic acids such as si-RNA and DNA, facilitating their transport directly to the tumor site while protecting the genetic material. Complexes combining SLNs, nucleic acid (pDNA or mRNA), the cationic peptide protamine (P), and polysaccharides like DX or HA were successfully created [180]. The research demonstrated that DOTAP-based cationic SLNs, due to their high stability and effective transfection, are promising as a non-viral vector for gene delivery, outperforming other lipid-based formulations with lower positive charges. These findings highlight the potential of SLNs as a promising approach to treating pancreatic cancer.

#### 6.2.3. Nanostructure Lipid Carriers

Nanostructured lipid carriers (NLCs) represent the second generation of solid lipid nanoparticles (SLNs), inheriting the benefits of SLNs while offering additional advantages. Studies have found that SLNs face challenges like the unintentional formation of different colloidal structures (e.g., micelles, liposomes) and drug nanocrystals during production and stability issues due to the complex physical states of lipids, leading to problems like gelation, drug expulsion, and particle size growth during storage or administration [181]. Particularly, SLNs are formulated with similar solid lipids that form a dense, orderly crystal network, which can limit the space available for drug molecules and reduce drug loading capacity. These lipids can undergo polymorphic transitions in favor of a more stable, low-energy crystalline form, potentially leading to drug leakage from the SLN matrix and affecting their stability, which can have profound stability implications [182]. NLCs, on the other hand, are composed of both solid and liquid lipids, creating a less dense structure and more disorganized structure without crystallizing, allowing NLCs to hold more drug molecules and reducing the risk of expulsion [183]. Depending on the nature of the solid and liquid lipid mixture, different types of NLCs can be obtained, including the imperfect structured solid matrix, the structureless solid amorphous matrix, and the multiple oils in solid fat in water (O/F/W) type.

Recent studies have demonstrated that NLCs perform more exceptionally than conventional formulations in delivering anticancer drugs and therapeutic-related hydrophobic peptides and proteins, particularly in treating PDAC. An NLC-based formulation for co-delivering gemcitabine and baicalein using HA as the targeting moiety, with the therapeutic agents being held in the core and HA covering the surface of the NLC shell, was successfully created [184]. The encapsulation efficiency of GEM and BCL reached 85.1% and 82.9%, respectively. In tests using mice models bearing human PDAC (AsPC-1 cells) tumors, the HA-targeted group demonstrated a superior antitumor effect compared to the free drug groups and the untargeted NLC group. As HA is abundantly present in the ECM of pancreatic tumors, targeting therapeutic agents through HA can effectively penetrate the tumor stroma. It may even aid in reducing the elevated IFP associated with HA. Notably, both cationic and neutral NLCs can deliver negatively charged substances like RNAs and DNAs, providing possibilities for gene therapies. Several studies show that NLC-based delivery systems are effective for delivering miRNAs for cancer treatment, including lung cancer and hepatocarcinoma, particularly when used in combination therapy [129,185]. These delivery systems exhibit a relatively safe profile, with low occurrences of adverse effects. A versatile NLC-based delivery system has been fabricated, utilizing an LHRH analog as a targeting agent, to transport doxorubicin or paclitaxel along with siRNAs targeting MRP1 mRNA and BCL2 mRNA to lung cancer cells overexpressing LHRH receptors. The NLC system successfully achieved targeted delivery of doxorubicin to cell nuclei while simultaneously confining siRNAs to the cytoplasm. This dual-action approach maximized drug cytotoxicity of anticancer drugs by inhibiting drug efflux pumps and cellular antiapoptotic defense via siRNAs, showing significant antitumor activity by NLC-based delivery systems compared to untargeted groups or unbound drugs [129]. These findings offer valuable insights for the design of more effective cancer treatments, specifically for PDAC.

#### 6.2.4. Lipid–Polymer Hybrid Nanoparticles 

Lipid–polymer hybrid nanoparticles were developed to combine the advantages of both polymeric nanoparticles and liposomes. They come in various types, each with specific advantages for different applications. One type is the polymer-core lipid-shell hybrid nanoparticles, which have a partial polymer core covered by a lipid shell. They can hold hydrophobic and hydrophilic drugs, making them suitable for anticancer therapeutics by co-delivering drug-resistance inhibitors and chemotherapeutic drugs [186]. Another type is the core-shell hollow lipid–polymer–lipid hybrid nanoparticles, which have a hollow core with cationic lipid layers. They possess the features of both lipoplexes and PLGA nanoparticles. They can efficiently capture anionic drugs, enabling the co-delivery of negatively charged nucleic acids such as siRNAs and small drugs to overcome drug resistance. Lastly, the polymer-caged liposome hybrid nanoparticles have polymer coatings on the surface of liposomes, which enhances stability and controls the drug release profile. Despite the versatility of hybrid nanoparticles, they possess common attributes, including a polymeric core that is both hydrophobic and hydrophilic for drug encapsulation, a biocompatible lipid shell encompassing the polymeric core, and an outer component comprising a lipid-PEG that is enveloped by a lipid layer. This structural design enhances drug loading and steric stabilization, prolongs in vivo circulation time, prevents immune recognition, and enhances active targeting, thereby enabling their potential use in anticancer therapeutics [186,187].

Several studies have demonstrated the feasibility of using lipid–polymer hybrid nanoparticles for PDAC treatment. Hu et al. successfully created hybrid nanoparticles by conjugating an anti-CEA half-antibody with a lipid–polymer structure. The maleimide-thiol coupling reaction was employed to attach the anti-CEA half-antibody to the lipid-PEG end. The hybrid nanoparticles have exhibited remarkable specificity towards PDAC cells expressing CEA, like BxPC-3 cells, while demonstrating marginal cellular uptake in non-CEA-expressing cells, such as XPA-3. The lipid shell and anti-CEA agents were securely attached to the polymeric core, indicating a highly selective and stable delivery mechanism that may aid in reducing off-target cytotoxicity in non-targeting organs. Furthermore, the targeting formulation has demonstrated more than a twofold increase in therapeutic efficacy, which could have significant implications for cancer treatment [188]. A recent study utilized modified nanoparticles to deliver two drugs, si-HIF1α and Gemcitabine, to PDAC tumor cells. The researchers employed PEGylated lipid bilayer-coated nanoparticles modified with ε-polylysine co-polymer, which possesses a positive charge for attaching ionic si-HIF1α on the surface. The hydrophilic core of the nanoparticles could retain gemcitabine, while the lipid bilayer prevented drug leakage. Furthermore, the surface charge of the nanoparticles could be flipped to prevent particle aggregation and degradation of siRNA, which also reduces serum protein absorption in vivo. The findings indicate that compared to the formulation without lipid cover, LENP-Gem-siHIF1α exhibited superior inhibitory effects against tumor growth, a better ability to avoid innate immune responses, and improved stability and prolonged circulation time in the bloodstream [189]. The use of hybrid nanoparticles has shown promise in PDAC immunotherapy. Researchers have developed a new method to fight PDAC using a combination of hybrid nanoparticles and chemotherapy. They incorporated phospholipid-indoximod (PL-IND) nanovesicles into a lipid bilayer surrounding mesoporous silica nanoparticles (MSNP) loaded with oxaliplatin (OX). This system induces both innate and adaptive anti-PDAC immunity by causing immunogenic cell death and inhibiting the immunosuppressive indoleamine 2,3-dioxygenase (IDO) pathway. This results in excellent tumor reduction or even elimination by recruiting cytotoxic T lymphocytes and reducing the presence of immunosuppressive Foxp3+ T cells [190].

### 6.3. Polymeric Nanoparticles

Both synthetic and naturally sourced polymeric materials can be utilized to fabricate polymer nanoparticles. Common synthetic materials used include PLGA, PCL, PEG, poly-n(cyanoacrylate), cyclodextrins, N-(2-hydroxypropyl) methacrylamide (HPMA), and polyamidoamine (PAMAM), similar to those needed for microparticles. Natural materials like gelatin, chitosan, alginate, dextran, heparin, collagen, albumin, and polyhydroxyalkanoates (PHAs), though potentially harder to source and extract, offer advantages like better bioavailability and biodegradability. As a result, they are overall less toxic and easier to eliminate from the body [191]. Polymers have demonstrated great versatility in creating nanoparticle formulations. They can adopt various structures, from traditional linear forms as polymer–drug conjugates to spherical configurations like polymeric micelles [191]. Self-assembled di-block copolymers, which create polymersomes, can encapsulate gemcitabine and erlotinib and be responsive to hypoxia, adapting to the oxygen-deficient microenvironment of PDAC. Additionally, immune-friendly nanocarriers made of polydopamine and Pluronic F127 are designed for easy adjustment of particle size without altering their composition. Furthermore, polymers can hybridize with other materials to create intricate complexes [192].

Besides Onivye, Abraxane stands as one of the only two FDA-approved nanoformulations currently used in clinical practice for the treatment of PDAC [167]. Abraxane, or nab-paclitaxel, is an albumin-stabilized nanomedicine that received FDA approval in 2013 for the first-line treatment of metastatic pancreatic cancer combined with gemcitabine [193]. Animal study outcomes demonstrated that nab-paclitaxel alone and nab-paclitaxel with gemcitabine significantly reduced the desmoplastic stroma in human tumor xenografted mouse models, evidenced by the immunohistochemical assay of collagen type 1 fibers. In contrast, tumors treated with only gemcitabine exhibited dense stroma, similar to the vehicle-treated group. In addition, stroma-depleted tumors that received combination therapy also showed dilated blood vessels and a 3-fold increase in mNestin, an angiogenesis marker of dividing endothelial cells. The 2.8-fold increase in intratumoral gemcitabine concentration in the combination therapy cohort suggests that vascularization can significantly improve the delivery of gemcitabine to the tumor site [194]. 

In a subsequent study, Frese et al. investigated the intratumoral concentrations of gemcitabine’s inactive (dFdU) and active (dFdCTP) metabolites, as well as the prodrug form (dFdC). They found that nab-paclitaxel administration significantly increased the levels of dFdC and dFdCTP, while the paclitaxel levels remained similar to those in the group treated with nab-paclitaxel alone. Further analysis revealed that paclitaxel and nab-paclitaxel could significantly lower the level of cytidine deaminase protein, which usually inactivates dFdC into dFdU, by introducing reactive oxygen species. Therefore, the regimen ultimately stabilizes the intratumoral accumulation of gemcitabine, even though the overall drug delivery did not change [195]. In a phase III study that incorporated 861 patients with metastatic PDAC, the effectiveness and safety of combining gemcitabine with nab-paclitaxel and using gemcitabine alone were evaluated and compared. Nab-paclitaxel/gemcitabine significantly outperformed gemcitabine alone in terms of median overall survival (8.5 months vs. 6.7 months), median progression-free survival (5.5 vs. 3.7 months), and response rate (23% vs. 7%) [196]. When combined, nab-paclitaxel and gemcitabine may be a helpful regimen to reduce further cancerous cell migration brought on by EMT from stromal cells and to improve treatment efficacy, particularly in cases of mPDAC that are gemcitabine-resistant [197]. 

#### 6.3.1. Dendrimers

Dendrimers are spherical and three-dimensional structures with a highly branched, multi-layered polymer architecture. The surface of these dendritic molecules can be readily modified with functional groups, which enables enhanced drug targeting and controlled release of the therapeutic agent. Drugs can be incorporated via mechanisms such as physical encapsulation within internal cavities, electrostatic bonding, and covalent conjugating between the drugs and functional groups on the dendrimer surface. The method used affects the release pattern of the drugs. For instance, drugs that are physically enclosed in the internal cavities of dendrimers are typically retained by hydrogen bond formation or hydrophobic and lipophilic interactions between nitrogen or oxygen atoms and the hydrophobic cavities. Ionizable therapeutic agents, on the other hand, can quickly form complexes with the terminal -NH2 and -COOH groups on the dendrimer surface through electrostatic interactions [168]. By encapsulating drugs in these ways, they can be released and controlled through changes in the physical environment, such as pH, temperature, etc. Drugs stabilized by covalent bonding can be released through in vivo degradation in the presence of enzymes or chemicals. This technique allows for better control over drug release and targeting specific cells.

Dendrimers can be hydrophilic, hydrophobic, amphiphilic, biodegradable, or glycodendrimers, and their functional properties vary accordingly [198]. They are categorized into several structural types, including PAMAM, PAMAMOS, PPI, and others [168]. Among these, PAMAM is notably well-studied and extensively used, including a commercially available type, Starburst^®^ [198]. A novel approach for treating PDAC has been developed using multifunctional PEGylated PAMAM dendrimers. These dendrimers are loaded with gemcitabine and conjugated to the anti-Flt-1 antibody, targeting the Flt-1 (a VEGF receptor) on Flt-1 positive PDAC cells like CFPAC-1. This targeting enhances the cellular uptake and therapeutic outcome of gemcitabine. In CFPAC-1 xenograft mouse models, this formulation showed a 30–50% inhibition of tumor growth, revealing a significant improvement compared to the minor changes by the untargeted dendrimer and gemcitabine solution [199]. Interestingly, a study by Huang et al. found that peptide dendrimers can be used as potentiators to enhance the accumulation of therapeutic agents. They synthesized polylysine dendrimers with tris(2-aminoethyl) amine (TAEA) as the core to explore their potentiating efficacy. The TAEA-K3K6R12 dendrimers, conjugated with DMA, could temporarily mask the positive surface charge. In an acidic tumor environment, the DMA groups were hydrolyzed, thus restoring their positively charged nature and facilitating the internalization of free doxorubicin or gemcitabine. The potentiator dendrimers remained neutral in blood circulation, effectively reducing cytotoxicity from drug accumulation in healthy organs. The group of mice that received both TAEA-K3K6R12-DMA and GEM had a significant reduction in mean tumor volume and weight compared to the group that only received GEM, with reductions of 86.5% and 87.1%, respectively [200]. This study provided promising insights for using modified dendrimers to enhance therapeutic agent uptake without the challenges of low drug loading/encapsulation that may be associated with other nanoparticle formulations.

#### 6.3.2. Nanospheres

Nanospheres are spherical structures made up of a dense solid matrix. These structures can either adsorb drugs onto their surface or co-precipitate drugs inside the polymeric matrix. Nanospheres are a subtype of polymeric nanoparticles and can be fabricated with natural and synthetic polymers, diblock or multiblock copolymers, etc. [201] They can encapsulate drugs, imaging agents, genetic materials, etc., making them versatile and practical in various fields, including drug and gene delivery, bio-imaging, and diagnosis [202]. 

In a research conducted by Rong et al., nanospheres were explored for diagnosis purposes using chitosan and poly(acrylic acid) doped with various metal ions, specifically Cu^2+^, Pb^2+^, Cd^2+^, and Zn^2+^. These metal ions were selected based on their distinct peak potentials. The nanosphere complexes, known as Cu-chitosan-poly(acrylic acid) or Cu-CP, Pb-CP, Cd-CP, and Zn-CP, were then combined with anti-CEA, anti-CA19-9, anti-CA125, and anti-CA242, respectively, to identify biomarkers for PDAC using electrochemical measurements. The study’s findings revealed that this approach produced highly accurate and precise results, closely resembling those obtained through the conventional Enzyme-Linked Immunosorbent Assay (ELISA) method.^3^

This innovative strategy has been tried to synthesize using different materials like albumin or PLGA as a delivery platform for PDAC standard treatment drugs like gemcitabine [203]. Li et al. developed gemcitabine-loaded bovine serum albumin nanospheres against pancreatic cancer cells. The optimized particle size group, which ranged from 50 nm to 200 nm, displayed uniform particle size, high drug loadings up to 11.25%, and encapsulation rates up to 82.92%. These results suggest that a particle size range of 200 nm is optimal for improved drug loading and encapsulation, while albumin was found to enhance these two parameters compared to PLGA in the synthesis of nanospheres. Moreover, the optimized particle size group exhibited relatively low IC50 against BxPC-3 cells compared to the free gemcitabine group [204]. Several studies have revealed the possibility of using nanospheres for theranostic purposes due to their versatility. A later study has developed advanced magnetic albumin nanospheres. These nanospheres have a core-shell structure where the outer shell is made of albumin. The interior core contains Fe_3_O_4_ magnetic nanoparticles (MNPs) along with gemcitabine. Furthermore, the surface of these nanospheres is functionalized with the anti-EGFR monoclonal antibody C225, making them a multifunctional delivery system for diagnosis and targeting treatment simultaneously. C225 and MNPs can target specific cells when an external magnetic field is employed. This dual-targeted approach proved to be exceptionally efficacious, inhibiting cell proliferation amounting to 85.18% and apoptotic rates of 49.31% against AsPC-1 cells. The system could also significantly lower MRI T2 values, offering an alternative approach for diagnosing early EGFR-positive PDAC noninvasively. This approach demonstrated superiority over both the single-targeted and non-targeted groups [204]. Tumor-associated TK1 mRNA-responsive DNA nanospheres (DNA-NS) that encapsulate doxorubicin were purposed for tumor detection and chemotherapy. These nanospheres were highly resilient to nuclease breakdown and are uniquely reactive to TK1 mRNA overexpression in malignant cells. They also have demonstrated superb therapeutic efficacy due to the ability to bypass the drug resistance of tumor cells [205].

Other polymeric materials were also used to formulate nanoparticles. For instance, vitamin E succinate-gemcitabine conjugate-loaded Pluronic^®^ was successfully developed and investigated for the intracellular delivery of the anticancer drug to pancreatic cancer cells [206]. 

### 6.4. Metal Nanoparticles

Technological advances such as nanotechnology have been introduced in cancer treatment and diagnostics to overcome the limitations of traditional types of treatments. Metal nanoparticles (MNPs), as nanosized particles, have found diverse applications in the diagnosis and treatment of cancer. Research indicates that these metal particles can modulate the activity of specific intracellular and extracellular signaling proteins, thereby influencing key processes like angiogenesis, metastasis, and inflammation [207]. By inducing apoptosis and cell cycle arrest, MNPs can limit or halt these processes. Importantly, when combined with other anticancer treatments, MNPs have the potential to significantly enhance their effectiveness, making them a valuable area of research in cancer treatment. 

Various types of methods were used for manufacturing MNPs. For instance, gold nanoparticles (Au NPs) were prepared in pure distilled water using a nanosecond Nd: YAG laser with a constant laser energy of 100 mJ and an ablation time of 5 or 10 min [208]. Using a transmission electron microscope (TEM) and UV-visible spectrophotometer analysis, the researchers investigated the structure and linear optical properties of the Au NPs. The TEM measurements revealed that the size of the Au NPs ranged from 20.3 to 14.1 nm, depending on the laser ablation time. The z-scan technique was employed to examine the nonlinear refractive index and nonlinear absorption coefficient of the Au NPs. The Au NP samples were irradiated at different excitation wavelengths ranging from 740 to 820 nm and at different average powers ranging from 0.8 to 1.6 W. The research showed that the Au NPs demonstrated a reverse saturable absorption (RSA) behavior that increased as the excitation wavelength and/or incident laser power increased. Furthermore, the Au NPs acted as a self-defocusing material whenever the excitation wavelength or incident power was modified.

Different types of metal nanoparticles can be prepared either alone or in combination with conventional chemotherapeutics to improve multifaceted anticancer activities. For instance, bio-engineered metallic nanoparticles can be designed as potential carriers of cancer vaccines. Spherical nanoparticles are preferred due to their easy cellular penetration in various biomedical applications. Functionalized nanoparticles are being developed to deliver nutraceuticals at targeted sites to combat oncological malignancies. However, nanoparticles have been reported to cause toxic effects after administration to the human body, mainly by mediating oxidative damage. To overcome this toxicity, plant-derived edible nanoparticles could be considered. Plants have the potential to be produced on a large scale, making them suitable for drug delivery applications [209].

### 6.5. Magnetic Nanoparticles 

Magnetic Nanoparticles (MNPs) have been developed as a means of facilitating both imaging and drug delivery, often in conjunction with other therapeutic agents [210,211]. Most of these nanoparticles range in size from 50 to 200 nm, with those measuring no greater than 100 nm demonstrating the ability to exhibit superparamagnetism [212]. Superparamagnetic iron oxide nanoparticles (SPIONs) have been widely explored as MRI contrast agents [193]. Specifically, ultrasmall SPIONs, ranging in size from 20 to 50 nm, are particularly adept at penetrating tumors and entering cancer cells while evading phagocytosis, which helps prolong their circulation time [213]. Furthermore, these nanoparticles are capable of producing heat when exposed to magnetic fields, thereby facilitating magnetic hyperthermia treatment. Jiang et al. have successfully fabricated sub-50 nm multifunctional superparamagnetic nanospheres with surface modification using LyP-1 to target p32 expressing pancreatic cancer cells precisely. The proposed Fe_3_O_4_@SiO_2_-FITC@mSiO_2_–LyP-1 nanospheres were synthesized by covering iron oxide nanoparticles with a layer of fluorescein-labeled silica and then immobilizing LyP-1 through the click reaction between the maleimide group on LyP-1 and thiol group on the nanospheres. The results of a tissue distribution study revealed that these nanospheres could accumulate in tumor tissues and overlap with p32, while there was no significant fluorescence observed in the non-targeted group or healthy organs such as the liver, spleen, heart, lung, or kidney. After one hour of administration of the targeted nanospheres, a significant decrease in signal on MR images in the tumor area was observed, which remained in the tumor for 24 h. In contrast, no significant signal change was observed in the non-targeted group, demonstrating the selectivity achieved by immobilized LyP-1 and the superparamagnetic properties of the iron oxide nanoparticles [214].

A set of studies explored the application of magnetic nanoparticles in magnetic hyperthermia therapy, particularly when used in conjunction with other therapeutic agents. In a xenograft murine model of pancreatic cancer, the expression of *CALR* gene, a marker of the immune response, significantly increased in cells containing MNPs. Additionally, magnetic hyperthermia (MH) treatment led to a notable accumulation of magnetic nanoparticles in the inner regions of the tumor. These results indicate that magnetic hyperthermia has the potential to activate the immune response and enhance particle internalization when an AMF is applied. One group proposed attaching gemcitabine to dextran-coated Fe_2_O_3_ magnetic particles via disulfide bonds to improve cell targeting and uptake in combination with magnetic hyperthermia. Significant improvements in cytotoxicity were observed in the in vitro study with PDAC cells BxPC-3 and MiaPaca-2, particularly against gemcitabine-resistant PANC-1 when an alternating magnetic field (AMF) was applied [212]. These findings indicate the potential synergistic therapeutic effects of combining magnetic hyperthermia and anticancer drugs.

### 6.6. Cancer Targeting 

Delivery of therapeutics specifically to cancer cells allows for solving two main tasks: (1) Enhancing the anticancer activity of the delivered drug(s) or other biologically active substances. In contrast to non-targeted delivery, when the delivered substance is distributed more or less evenly throughout the body, its targeted delivery directly to the affected organ, tissue, or cell reduces the overall required concentration of the delivered substance, thereby increasing the effectiveness of treatment. (2) Protecting healthy organs and tissues from the damage caused by the delivered (usually highly toxic) anticancer agent, therefore limiting adverse side effects of the treatment. Previously, we subdivided all possible cancer targeting approaches into two general categories, passive and active targeting, and discussed various variants of both strategies in detail [215]. From the point of view of the current paper, we will discuss two major approaches that are the most relevant for the treatment of PDAC: the enhanced permeability and retention effect and active targeting by targeting ligands attached to the surface of nanoparticles that can specifically bind to the receptors or other molecules on the surface of PDAC cells.

#### 6.6.1. Enhanced Permeability and Retention (EPR) Effect

**Figure 7:**
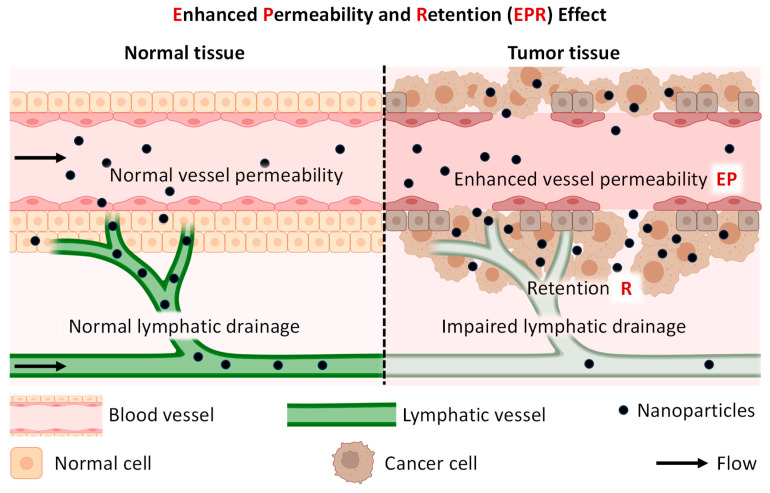
Schematic representation of the enhanced permeability and retention (EPR) effect. Redrawn from [218]. The red letters highlight the first letters in the Enhanced Permeability and Retention (EPR) effect abbreviations: EP stands for Enhanced Permeability, and R stands for Retention.

Enhanced accumulation of macromolecules in solid tumors and its mechanisms were first reported by Matsumura and Maeda in 1986 and termed the “Enhanced Permeability and Retention (EPR) effect” [216]. The EPR effect is a phenomenon that occurs when the blood vessels surrounding a relatively large tumor become highly permeable while there is minimal lymphatic drainage from the tumor (Figure 7). This combination results in increased penetration of nanoparticles into the tumor and insufficient washing away of them by lymph. As a result, the particles accumulate and get retained in the tumor. As substances with high molecular weight, nanoparticles are naturally susceptible to this effect and, therefore, will passively accumulate in solid tumors. However, it has been observed that the use of enhanced EPR effects to transport nanomedicines to tumor sites is not consistently effective and is instead dependent on specific characteristics of the tumor and its physiological conditions [217]. Factors such as the degree of angiogenesis and lymphatic development, the amount of pericyte coverage in the tumor vasculature, the density of the tumor stroma, and intratumoral pressure are principal contributors to the variability in EPR effects. Understanding these aspects resulted in the creation of active targeting approaches, which currently represent the mainstream in cancer-targeted drug delivery under development and clinical trials.

#### 6.6.2. Active Targeting

**Figure 8:**
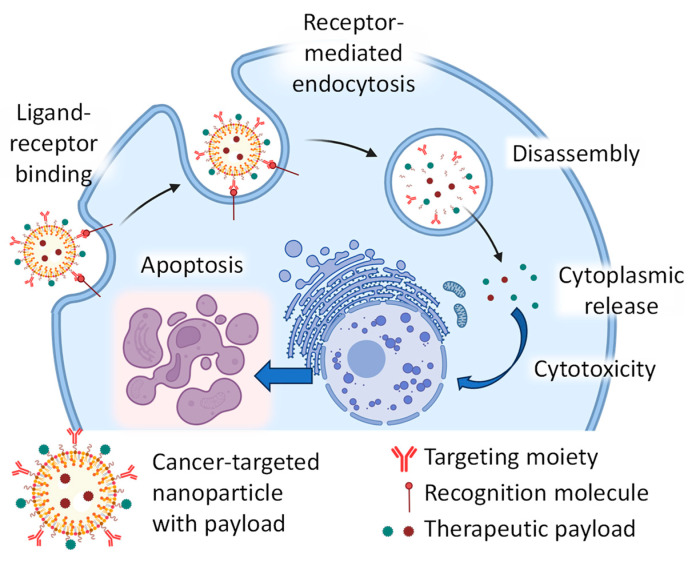
Simplified schematic representation of uptake of tumor-targeted nanoparticles by cancer cells. The nanoparticle surface is decorated by a targeting ligand and contains a therapeutic payload(s). Binding the nanoparticle to a targeted receptor overexpressed on the cancer cell’s surface initiates its internalization through receptor-mediated endocytosis. Once inside, the nanoparticle system is broken down within the endosome, typically after fusing with lysosomes. After the escape of cytotoxic payload into the cytoplasm, cancer cell death through apoptosis is induced.

Extensive research has focused on enhancing nanoparticle specificity, stability, and efficacy in cellular uptake and retention. These efforts ultimately have led to the development of “active targeting” and optimization of the therapeutic potential of drugs. Although several distinct approaches have been developed, cancer-targeted nanoparticles are often designed to attach to targeting moieties such as aptamers, proteins, peptides, antibodies, antibody fragments, receptor ligands, and other molecules. By binding their counterparts, such moieties initiate active penetration of nanoparticles with their cargo, specifically into cancer cells, mainly by endocytosis, disrupting the carriers inside the cellular cytoplasm and releasing active components (Figure 8). This specific targeting improves the accumulation and retention of nanomedicines within tumors, significantly reducing the side effects and off-target impacts on healthy organs often seen in traditional chemotherapy. Researchers are also developing stimuli-responsive nanoparticles that release drugs in response to specific triggers in the tumor microenvironment, such as pH changes, temperature shifts, enzyme activity, and hypoxia. External stimuli such as heat, ultrasound, light, and magnetic fields are frequently employed to aid in designing targeted nanoparticles.

### 6.7. Potential Recognition Molecules for Targeted Therapy of Pancreatic Cancer

**Figure 9:**
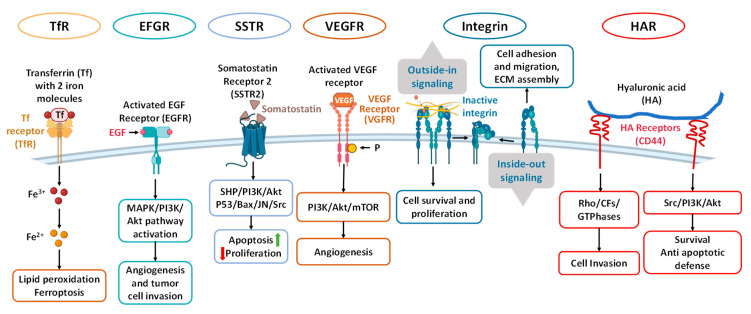
Potential recognition molecules for targeted therapy of pancreatic cancer.

Targeted therapy, which aims to selectively deliver therapeutic agents to cancer cells while sparing normal cells, has emerged as a promising approach for treating pancreatic cancer. One key aspect of targeted therapy is the use of recognition molecules that specifically bind to cancer cells, allowing for the delivery of therapeutic agents directly to the tumor site. In this comprehensive analysis, we will explore potential recognition molecules and their targets that can be used for targeted pancreatic cancer therapy (Figure 9). As shown below, interaction with these receptors and surface-bound molecules can, to a certain extent, initiate cell death in pancreatic cancer cells. However, the more robust approach should be based on active targeted delivery of more potent anticancer drugs to PDAC. Such an approach can be achieved by conjugating the ligands mentioned below to the surface of nanoparticles loaded with traditional, highly potent anticancer agents.

#### 6.7.1. Transferrin Receptor (TfR)

Transferrin receptors are abundantly expressed in PDAC malignant cells and significantly impact cell proliferation and cancer progression, especially by contributing to mitochondrial respiration and ROS production [219]. It was pointed out that 93% of pancreatic tumor cells exhibited positive (82%) or heterogeneous (11%) expression of transferrin receptors, demonstrated by immunohistochemical staining. Conversely, normal pancreas tissues were free of staining, indicating the deficiency of transferrin receptor expression [219]. The transferrin-bound iron was uptaken into cells via the transferrin receptor-mediated endocytosis pathway, making overexpressed transferrin receptors a desired target for delivering therapeutic agents to specific sites (Figure 9). A transferrin-mediated delivery system for p53 restoration and enhancing gemcitabine efficacy was developed [220]. In the study, liposomal nanoparticles loaded with wild-type human p53 (SGT-53) were formulated and evaluated. The nanoparticles were surface-modified with a single-chain antibody fragment (TfRscFv) for transferrin receptors. The group that received the TfRscFv-liposomal-SGT-53/gemcitabine combination treatment demonstrated a significantly longer median survival time of 37 days, compared to 29 days for the TfRscFv-liposomal-SGT-53 group and 30 days for the gemcitabine group, demonstrating the feasibility of using transferrin receptors as a gene delivery target in for treating PDAC. The first clinical trial involving human subjects in Phase I demonstrated that the utilization of nanoparticle p53 led to the restoration of p53 protein levels while achieving high drug accumulation at the tumor site and maintaining a relatively favorable safety profile [221]. Notably, the Phase II clinical trial involved a combination of nanoparticle p53 with gemcitabine/nab-paclitaxel, exhibiting a significant improvement in the median progression-free survival (mPFS) to 7.4 months in FOLFIRINOX refractory patients, compared to 3.1 months from the group that received the currently approved second-line therapy [222].

#### 6.7.2. Epidermal Growth Factor Receptor (EGFR)

Epidermal Growth Factor Receptor (EGFR) is crucial in cellular communication within normal and cancerous cells. It is prominently overexpressed in various cancers, including lung, colorectal, pancreatic, and breast [223]. In PDAC, reports indicate overexpression rates ranging from 30% to 89% [18]. As a tyrosine kinase receptor, this 170 kD glycoprotein consists of distinct domains: an extracellular portion for ligand attachment, a transmembrane segment, and an intracellular domain containing tyrosine residues (Figure 9). Upon binding to ligands like EGF or TGF-α, two receptor subunits dimerize, activating the tyrosine kinase domain. Consequently, tyrosine residues undergo phosphorylation, creating binding sites for other proteins. This event triggers downstream intracellular signaling via the RAS-MAPK and PI3K-AKT pathways [224]. Studies propose that pancreatic cancer initiation might involve multiple gene mutations. In cancer cells, elevated EGFR levels can activate other proto-oncogenes, such as cyclin D1 and COX-2. Moreover, evidence indicates that tumor-secreted agents in the microenvironment, like VEGF, IL-8, and FGF, can be significantly amplified by EGFR activation in neighboring cells. This potentiation contributes to tumor progression, facilitating angiogenesis and subsequent metastasis [224].

There are two primary strategies to impede EGFR activity and subsequently dismantle the signaling cascade, depending on the specific sites being targeted on the receptor [224]. A monoclonal antibody (mAb) hinders the extracellular binding site of EGFR 226,227. Due to the prolonged serum half-life, enhanced specificity, and dependable pharmacokinetic profile, the mAb retains substantial potential in EGFR targeting [224]. Another approach targets the intracellular tyrosine kinase domain with tyrosine kinase inhibitor (TKI). These TKIs interfere with the signaling pathway by countering ATP, inhibiting autophosphorylation. Erlotinib, combined with gemcitabine, is currently approved by the FDA as a therapeutic regimen for locally advanced and metastatic pancreatic cancer. This combined treatment approach manifests only a marginal increase in survival rates (6.24 months versus 5.91 months), comparable to gemcitabine monotherapy [225]. Findings from a Phase III trial have indicated that KRAS mutation might contribute to TKI resistance by consistently activating downstream signal transduction, independent of TK activation.

#### 6.7.3. Somatostatin Recept 6.6 or 2 (SSTR2)

Originally isolated from the hypothalamus, somatostatin (SST), or somatotropin release-inhibiting factor (SRIF), is a cyclic tetradecapeptide (SST-14) abundantly synthesized by neuroendocrine cells [226]. Along with the 28 amino acid variants (SST-28) with extension at the N-terminus, they are widely distributed in the body, including the central and peripheral nervous system, gastrointestinal tract, and various visceral organs. Depending on the site of secretion, SST has multiple biological functions and primarily acts as an endogenous growth inhibitor, regulating and proliferative activities of cells via paracrine or endocrine pathways [226].

SST is mediated by a family of five subtypes (SSTR 1–5) of seven transmembrane G-protein-coupled receptors [227]. Previous research suggested that SSTRs are present in various neuroendocrine tumors, including pituitary adenomas, pancreatic endocrine tumors, small cell lung cancers, medullary thyroid carcinomas, paragangliomas, carcinoids, and others [226,227]. Notably, SSTRs are also found on angiogenic tumor blood vessels, highlighting the potential as targets for cancer treatment. Clinical studies have shown that SST has tumor-suppressive effects on various cancers, including acromegaly, endocrine pancreatic cancer, and ectopic tumors like gastrinomas and VIP-producing tumors [228]. However, native SST has an extremely short half-life of 1–3 min in circulation, consequently a short duration of action and limited clinical application. The development of synthetic short peptide analogs has significantly extended their half-life and improved therapeutic outcomes. Unlike the endogenous SSTR agonists with high binding affinity to all the five SSTR subtypes, the synthetic peptide analogs show selective binding affinity towards SSTR 2, 3, and 5, the most common subtypes found on tumor cells [229]. Octreotide, a widely used octapeptide analog with a prolonged half-life of 2 h, shows a high affinity for SSTR2 and SSTR5, making it a promising targeting agent for SSTR-positive tumors [230]. 

Different expressions of SSTR2 in PDAC tissues and cell lines were found, indicating the potential for receptor-mediated chemotherapy [229]. Various studies report that at least one SSTR subtype mRNA exists in many PDAC cells. SSTR2 mRNA, the most common subtype, has been detected in several human PDAC cell lines, including CAPAN-1, CAPAN-2, PANC-1, BxPC-3, AsPC-1, CFPAC-1, and MiaPaca-2 [231]. Kikutsuji et al. found SSTR1 and 2 in at least 7 out of 10 exocrine pancreatic cancer tissues [232]. Further, it was reported that in a study of 108 PDAC patients, 81.5% (88/108) expressed SSTR 2 mRNA in their cancerous tissues. Additionally, SSTR-3 mRNA was found in 69.4% (75/108) of these tissues, while SSTR-5 mRNA expression was observed in 13.0% (14/108). This research underscores the potential for targeted therapies based on SSTR expression in pancreatic cancer [233]. 

A study highlighted neuroendocrine differentiation in pancreatic cancer cells through the expression of SSTR2, a marker for neuroendocrine tumor cells. Unique morphological patterns were identified in Panc-1 and MiaPaca-2, with high SSTR2 expression observed particularly in polymorphic MiaPaca-2 and small cells (morula) of Panc-1, evidenced by immunohistochemistry [234]. Further research has consistently shown that PDAC cell lines, including Panc-1, CFPAC-1, Capan-1, and Capan-2, express one or more SSTR subtypes, validated by SST analog RC-160 binding assay [235]. 

#### 6.7.4. Vascular Endothelial Growth Factor Receptor (VEGFR)

The VEGF receptor commonly interacts with various ligands, including VEGF-A, -B, -C, -D, and placenta growth factor (PLGF) [236]. Each subtype binds specifically to distinct receptors, regulating the processes of vasculogenesis, angiogenesis, and lymphangiogenesis (Figure 9). VEGF receptors are frequently elevated in various malignancies, including ovarian, colorectal, gastric, pancreatic, breast, prostate, small-cell lung, melanoma, cervical, and thyroid [236]. Like receptor tyrosine kinases (RTKs), ligand-activated VEGF receptors activate cellular signaling pathways by autophosphorylating their tyrosine kinase domains. Functioning as an endothelial cell mitogen, VEGF promotes tumor proliferation and metastasis, ultimately resulting in unfavorable prognostic outcomes. The upregulation of VEGF has been reported in over 90% of pancreatic cancer cells, thereby a potential target when formulating novel therapeutic approaches [237]. Small molecule RTK inhibitor Sunitinib can effectively impede VEGF receptor function and disrupt downstream signaling transduction by competitively binding to the ATP-binding site. The first FDA-approved anti-VEGF monoclonal antibody, Bevacizumab, operates by obstructing the interaction between VEGF-A and the VEGF receptor-2 within PDAC cells [236,237]. This intervention effectively hinders the ERK, PI3K/Akt, and p38/MAPK signaling cascades. Consequently, the reduction in cell–cell adhesion molecules ZO-1/ZO-2 by VEGF-A is impeded, which in turn may mitigate carcinoma cell migration. Nonetheless, while the administration of Bevacizumab in combination with other chemotherapy drugs, such as gemcitabine plus erlotinib, may enhance progression-free survival, the enhancement in overall survival remains modest [238]. These results show that resistance to Bevacizumab could potentially develop throughout treatment. Research findings indicate that pro-inflammatory factors, such as IL-1α and IL-1β, are expressed at higher levels in the group exhibiting resistance to anti-VEGF treatment, indicating that the development of resistance may arise from the inflammatory response [239]. A recent study has demonstrated that chemotherapy may enhance the secretion of PLGF. Consequently, increased PLGF can bind to VEGF co-receptors, neuropilin-1 (NRP-1) and -2 (NRP-2). This interaction subsequently activates cancer-associated fibroblasts (CAFs), which generate ECM, contributing to the formation of the desmoplastic stroma [240]. An anti-PIGF/VEGF agent within the therapeutic regimen is imperative to counteract the collagen matrix formation in PDAC, holding the potential to amplify the antitumor efficacy of chemotherapy significantly.

#### 6.7.5. Integrin

Integrin is evaluated in many studies and is recognized to be highly associated with tumor growth by regulating cell functions. It is a group of heterodimeric transmembrane glycoproteins consisting of combinations of 18 α subunits and eight β subunits. These subunits are connected by non-covalent bonding and result in 24 different combinations. Integrins are critical in cell adhesion and interaction with the ECM and adjacent or neighboring cells [241]. To be functional, integrins must change their confirmation from bent closed inactive to extended open active. Integrins have two unique mechanisms for transmitting signals in both directions (Figure 9), known as “inside-out” and “outside-in,” indicating that they can be activated by intracellular or extracellular signals, which leads to different consequences [241]. In the case of “inside-out” activation, intracellular activators such as talin and kindlin bind to the cytoplasmic tails of integrins, leading to a conformational change to its active form with an extended structure and open tails. This active phase provides high affinity towards ECM ligands, followed by enhancement of the adhesion between integrin and ECM, allowing force transmission needed by cell migration, invasion, ECM remodeling, and matrix assembly. On the other hand, “outside-in” activation occurs when extracellular signals bind to the integrin extracellular domain, causing a conformational change to its active form. This change, in turn, leads to integrin clustering and downstream effector initiation 264. Importantly, the bidirectional signaling pathways are closely linked, regulating cell polarity, survival, proliferation, gene expression, and cytoskeleton organization. 

Targeting tumor vasculature has distinct advantages over other therapeutic approaches, such as direct contact with vascular endothelial cells or blood vessels and a lower likelihood of inducing drug resistance due to the high gene stability of endothelial cells [242]. Studies have shown that eight different integrins can recognize a targeting agent RGD (Arg-Gly-Asp) peptides, and five of them are from the αV subfamily [243]. Among them, αVβ3 and αvβ5 are expressed abundantly on activated endothelial cells of pancreatic tumor vasculature. Around 58% of PDAC cases were demonstrated with overexpression of αVβ3, which was linked to lymph node metastasis and the activation of MMP-2 [244]. Meanwhile, αvβ5 works in corporation with αVβ3 through TGF-β mediated dimerization to activate the focal adhesion kinase (FAK)–steroid receptor coactivator (Src) pathway, leading to the promotion of angiogenesis and consequently tumor progression 265. The improved form iRGD, a cyclic peptide of nine amino acids, including the RGD motif, was designed to target the tumor vasculature and penetrate tumor tissues. After binding to the tumor vasculature, the peptide is cleaved by proteases, revealing an activated C-terminal [243]. The cleaved iRGD then binds to NRP-1, which is overexpressed in about 45.8% of clinical PDAC lesions, enabling the targeted delivery of therapeutic agents to the inner areas of the tumor, thereby improving treatment efficacy. A series of studies focused on the utilization of iRGD. A simplified approach for co-delivering iRGD and various therapeutic drugs, including doxorubicin, nab-paclitaxel, doxorubicin liposomes, and trastuzumab into five tumor models, two of which were PDAC models was proposed [245]. The combination led to a significant increase in drug accumulation at tumor sites while achieving equivalent or even better therapeutic efficacy with a threefold lower dosage than the drug-alone therapies, suggesting reduced side effects. Later, an approach that co-delivered iRGD and free gemcitabine to PDAC murine models, which substantially enhanced the efficacy of gemcitabine and caused a significant shrinkage of tumor size compared to the gemcitabine monotherapy group, was developed [245].

A significant limitation of targeting the integrin ligand binding site is that molecular antagonists often activate integrin signaling, restricting the clinical effectiveness. A phase I trial of the ProAgio, an anti-αvβ3 protein, is ongoing for patients with PDAC or other solid tumors. The approach was aimed at binding the protein to a novel site on αvβ3 protein, recruiting and activating caspase eight at the cytoplasmic tails of integrin β3, thereby inducing apoptosis in endothelial blood vessels cell and stroma-supporting fibroblasts, leading to a reduction in angiogenesis and tumor growth [246]. 

#### 6.7.6. Hyaluronic Acid Receptors 

The expression of HA is highly upregulated in most PDAC samples, mainly in connective tissues and tumor boundaries, exhibiting an enormous increase compared to the normal pancreas [19]. It is a significant component of the ECM and PDAC tumor stroma. In recent years, research has focused on targeting HA due to its vital role in enhancing tumor chemoresistance and metastasis [19]. Previous studies have proposed and analyzed potential therapeutic methods to reduce the levels of stroma HA by either inhibiting its synthesis or enzymatically depleting its amount. One such method involves using 4-methylumbelliferone (4-MU), an HA synthesis inhibitor [247]. The effects of 4-MU treatment on Mia Paca-2 cells regarding cell proliferation, motility, invasion, and pericellular matrix containing HA were analyzed. The results indicated an inverse relationship between the pericellular matrix-to-cell ratio and the concentration of 4-MU. The treatment of 0.5 mM 4-MU for 72 h significantly reduced cell proliferation by 26.4% and suppressed cell migration and invasion by 14.7% and 22.7%, respectively, as shown by the wound healing and Matrigel invasion assays. Another study evaluated the HA production and cell migration capability by co-culturing PANC-1 and tumor stromal fibroblasts [248]. The co-culture system exhibited significant increases in HA and HAS3 mRNA. With 1000 uM treatment of 4-MU, 88% inhibition of HA synthesis was observed, while cell migration inhibition was seen at a concentration as low as 10 uM. The findings indicated that 4-MU can suppress the synthesis of HA by decreasing the levels of HAS3, reducing low-molecular-weight HA (LMW-HA) production, which facilitates cellular motility and invasion more robustly than high-molecular-weight HA (HMW-HA). These results were consistent with earlier studies on in vitro and in vivo models using KP1-NL cells [249]. Notably, the mechanisms by which 4-MU suppresses HA synthesis may vary among different cell types. Nagase’s study showed upregulation of HAS3, suggesting an alternative mechanism where the depletion of UDP-GlcUA, followed by the inhibition of HA synthesis by 4-MU, may be utilized. 

PEGylated human hyaluronidase (PEGPH20) was proposed as an enzyme that degrades HA in PDAC stroma. Given the promising results from phase I/II trials, phase III HALO-109–301 compared the effects of PEGPH20 in combination with nab-paclitaxel/gemcitabine to nab-paclitaxel/gemcitabine alone in untreated stage IV PDAC patients with high HA expression [250]. However, HALO 301 failed to meet its primary endpoint of OS at 11.2 months when used in combination compared to 11.5 months with nab-paclitaxel/gemcitabine alone, although a higher response rate was observed in the combination treatment group. Similar adverse outcomes were observed in two later clinical studies. In the SWOG S1313 phase IB/II study, mFOLFIRINOX was used both with and without PEGPH20. However, including PEGPH20 in the combination arm shortened the median OS due to increased grade 3 to 4 adverse effects [250]. The MORPHEUS Phase IB/II study compared atezolizumab plus PEGPH20 with mFOLFOX6 or gemcitabine plus nab-paclitaxel chemotherapy. The study showed a marginal increase in OS but a reduction in PFS in the PEGPH20/atezolizumab regimen compared to the chemotherapy group. These results indicated that targeting the desmoplastic stroma alone is insufficient for PDAC treatment, highlighting the need to focus on the immunosuppressive cells in the tumor microenvironment. Hakim et al. noted that tumor stroma may have multiple roles in metastasis. Besides being an obstacle impeding drug delivery, it may inhibit tumor development and advancement. Therefore, PEGPH20 can potentially facilitate tumor advancement by removing this barrier. Further research on therapeutic approaches needs a more thorough understanding of the intricate factors contributing to chemoresistance, the multifaceted functions of EMT, and the diverse roles tumor stroma plays in metastasis.

Studies have revealed that hyaluronan interactions with its receptors CD44s and Receptors for HA-Mediated Motility (RHAMM) trigger most HA signaling pathways, resulting in inflammations and tumorigenesis in PDAC cases [251]. Flow cytometry analysis studies have confirmed that CD44 levels in human PDAC cells, such as PANC-1, Mia Paca-2, BxPC-3, and AsPC-1, are over 100 times higher than in healthy pancreas cells 287. Moreover, CD44v5 and CD44v6 are exclusively expressed in PDAC cells and not frequently on IPMN, indicating a more advanced and invasive form of cancer. The binding of HA to CD44 variants has been shown to activate several signaling pathways, including the Rho ATPase, Ras/MEK/ERK (MAPK), Rac, and PIAK/Akt pathways, which can promote cytoskeletal transformation, cell migration, invasion, survival, proliferation, and drug resistance [19,251]. Moreover, RHAMM’s interaction with HA stimulates FAK, enhancing MAPK pathway activation and increasing cell motility. Approaches targeting HA receptors, such as RHAMM and CD44, involve blocking receptor-mediated signaling pathways or using overexpressed HA receptors for site-specific delivery of therapeutic agents [252]. The suppression of CD44 expression using shRNA in pancreatic cancer cells led to reduced cellular proliferation and migration, thereby impeding tumor growth and progression, consistent with decreased levels of p-ERK and p-AKT.290 Monoclonal antibodies against CD44 (anti-CD44), such as H4C4, can significantly attenuate the stem cell self-renewal genes Nanog, Sox-2, and Rex-1, as well as regulating STAT3 signaling, thereby inhibiting the post-radiation recurrence in mice xenografts [253].

A styrene-maleic acid (SMA) copolymer nanomicelle system with surface modification of HA conjugation that delivers 3,4-difluorobenzylidene curcumin (CDF) to CD44+ PDAC cancer stem-like cells (CSLCs) was developed [254]. Compared to the group treated by naked SMA-CDF, the cellular internalization was more pronounced in the MiaPaca-2 cells treated by the HA-conjugated nanomicelle system (HA-SMA-CDF). HA-SMA-CDF also performed better than the SMA-CDF in the cytotoxicity assay, with 1.75- and 2-fold lower IC_50_ in MiaPaca-2 and AsPC-1 cells, respectively. Moreover, the targeted group showed better anticancer efficacy in the CD44+ cells than in the CD44- group. The study provided consistent in vitro outcomes when utilizing HA-modified liposomes or nanoparticles to deliver dual drugs (doxorubicin/paclitaxel and quercetin/gemcitabine) to tumor cells overexpressing CD44. Notably, the surface modification of HA was found to be a great alternative to PEGylation as it can alter the surface charge of liposomes, allowing for more extended circulation of nanoparticles. Moreover, it overcomes the limitations of PEG presence, which hinders interaction between nanocarriers and cell surfaces [254]. These findings provide valuable insight into the molecular mechanisms of PDAC development, highlighting the potential of hyaluronan and its CD44s and RHAMM receptors as targets for PDAC treatments.

### 6.8. Nanoparticle-Based Drugs in Clinical Trials

Several phase 1 and 2 clinical trials designed to evaluate nanoparticle-based drugs for prostate cancer treatment are being conducted and completed (Table 1). Most of these trials tested protein-bound paclitaxel (nab-paclitaxel or Abraxane) in combination with traditional or novel anticancer drugs. In addition, superparamagnetic iron oxide magnetic nanoparticles were tested for tumor imaging. Moreover, a few other types of nanoparticle-bound drugs are also used, including lipid-based siRNA nanoparticles, novel curcumin/doxorubicin-encapsulating nanoparticles, and liposomal Irinotecan. It can be seen that despite numerous preclinical studies, only a tiny portion of nanomedicines reached clinical trials. Therefore, more efforts should be applied to design and test prostate cancer-targeted nanoscale-based delivery systems.

## 7. Conclusions and Future Perspectives

Early diagnosis and innovative therapies are crucial for improving the survival rates of patients with this highly lethal cancer. The application of biodegradable fluorescent targeted nanoparticles as imaging agents significantly enhances the accumulation of fluorescence in specific tumor cells in pre-cancerous pancreas lesions, offering new possibilities for early detection of PDAC. Some researchers suggest merging computer vision and biophotonics to gain more insights into tumor biology and aid decision-making for patients with pancreatic cancer [256]. “Surgomics” is a growing field that encompasses such efforts, and it represents a concept that aims to predict a patient’s surgical outcome using machine learning techniques on multimodal intraoperative data [257]. This approach involves utilizing high-quality annotations by medical experts to develop predictive models to assess morbidity, mortality, and long-term outcomes in surgery. Surgomics uses machine learning algorithms to analyze various aspects of the surgical process, such as bleeding events, instrument recognition, and anatomical structures, to improve the understanding and prediction of surgical outcomes. By extracting surgomics features as surgical process characteristics, it is possible to personalize surgical procedures and improve patient outcomes.

Continued research into nanoparticles that can specifically target and illuminate multiple early-stage cancerous markers may hold the potential for a significant step forward in precise diagnostic methods. Such advancements could significantly enhance the effectiveness of surgical and therapeutic interventions.

Following the FDA approval of Abraxane and Onivyde, the field of nanomedicine in PDAC treatment presents a large area for exploration and development, especially considering the effectiveness demonstrated by these two drugs. These two drugs have a relatively “simple” design, meaning they mainly depend on the passive targeting mechanisms for delivering a single drug. This highlights the significance of customizing the nanoparticle formulations by adjusting their physiochemical properties. It is essential to consider the pharmacokinetics and pharmacodynamics of the payload in specific tumors. Such tailored approaches are key to effectively navigating the complex tumor microenvironment in PDAC, enhancing the EPR effect, and offering the potential for delivering multiple drugs within a single delivery system, thereby increasing treatment efficacy and targeting accuracy. Research has indicated that no nanomedicines employing active targeting ligands have yet received clinical approval, mainly due to challenges like the “binding site barrier,” where nanomedicines are absorbed by peripheral tumor cells, and challenges in selecting appropriate patients for clinical trials [258]. Future research that combines tumor-penetrating peptides with these nanomedicines may provide valuable insights and improvements in targeting efficiency, overcoming current barriers, and enhancing the effectiveness of nanomedicine in PDAC treatment. Due to the PDAC’s heterogeneous nature, targeting cancer-related genes holds promise as an effective method to inhibit tumor proliferation and metastasis. However, traditional pharmacological inhibitors and small molecule drugs often fall short of expectations regarding many of these genes and proteins, considered “undruggable”. This challenge arises from the absence of specific ligand binding sites and target selectivity, often due to the close amino acid sequence homology these proteins share with others [27]. Delivering RNA interference agents via nanoparticles presents a promising alternative to target cancer-related genes, offering high selectivity and safety, the ability to transfer larger genes and customizable options.

Furthermore, in vivo trials with virus-like particle (VLP) vaccines have shown increased immune cell infiltration, potentially making immunotherapy more effective in PDAC’s immunosuppressive environment. This may overturn the immunotherapy landscape in PDAC, offering new hope where previous applications were limited. Collectively, the nanoparticle delivery system holds great potential in overcoming the limitations of traditional diagnostic and therapeutic approaches, with precision in diagnostics, augmented drug solubility, prolonged circulation time, improved targeting of tumors, and controlled release. Further research is essential to comprehensively understand the complex microenvironment and distinct pathological features at different stages of PDAC. This will enable the development of nanoparticle delivery systems that are not ‘one-size-fits-all’ but can be modified to target the varying tumor features. Such modification ensures that these systems meet the patient’s unique needs, providing a more tailored and potentially more effective treatment approach with minimized side effects. Importantly, it is vital to ensure the scalability of these formulations, making them accessible to a broader range of patients, thereby significantly enhancing the battle against this lethal disease.

## Figures and Tables

**Table 1:** Recent active and completed clinical trials involved the treatment of pancreatic cancer with nanoparticle-based drugs. Selected results of the search of clinicaltrials.gov (accessed on 10 February 2024) [255]. The list does not include canceled or withdrawn clinical trials.

#	NCT Number	Status	Interventions	Phases
1	NCT02336087	Active	Paclitaxel Albumin-Stabilized Nanoparticle Formulation combined with Gemcitabine, Metformin	1
2	NCT02562716	Completed	Paclitaxel Albumin-Stabilized Nanoparticle Formulation, Fluorouracil, Gemcitabine, Irinotecan, Oxaliplatin	2
3	NCT02194829	Completed	Nab-paclitaxel *, Gemcitabine	1, 2
4	NCT03410030	Completed	Paclitaxel protein-bound, Cisplatin, Gemcitabine	1, 2
5	NCT02707159	Completed	Drug: Nab paclitaxel, Gemcitabine	2
6	NCT02227940	Completed	Paclitaxel Albumin-Stabilized Nanoparticle Formulation, Ceritinib, Cisplatin, Gemcitabine	1
7	NCT00920023	Completed	Superparamagnetic Iron Oxide Magnetic Resonance Imaging	4
8	NCT02178436	Completed	Nab paclitaxel *, Gemcitabine, Selinexor	2
0	NCT02620865	Completed	Paclitaxel Albumin-Stabilized Nanoparticle Formulation, Biologicals: Aldesleukin, Antibody Therapy, Drugs: Fluorouracil, Gemcitabine, Irinotecan, Leucovorin Calcium, Oxaliplatin, Sargramostim	1 2
10	NCT01677559	Completed	Nab-Paclitaxel *	1
11	NCT03304210	Completed	PIPAC (Pressurized intraperitoneal aerosolized chemotherapy) with Abraxane	1
12	NCT03910387	Active	Nab-paclitaxel *, Gemcitabine, Telotristat Ethyl	2
13	NCT01161186	Completed	Nab-paclitaxel *, Gemcitabine, Capecitabine	1
14	NCT02333188	Completed	Paclitaxel Albumin-Stabilized Nanoparticle Formulation, Leucovorin, Irinotecan, Fluorouracil	1
15	NCT01437007	Completed	TKM-080301—stable nucleic acid–lipid particles (SNALPs) formulation of a siRNA against Polo-Like Kinase 1 (PLK1)	1
16	NCT04524702	Active	Nab-paclitaxel *, Gemcitabine, Hydroxychloroquine, Paricalcitol	2
17	NCT02394535	Completed	Nab-paclitaxel *, Capecitabine, Radiation Therapy	1
18	NCT03382340	Active	Curcumin/Doxorubicin-encapsulating Nanoparticle (IMX-110)	1 2
19	NCT02930902	Completed	Nab-paclitaxel *, Gemcitabine, Paricalcitol, Pembrolizumab	1
20	NCT02427841	Completed	Nab-paclitaxel *, Fluorouracil, Gemcitabine, Image Guided Radiation Therapy, Intensity-Modulated Radiation Therapy	2
21	NCT02231723	Completed	Nab-paclitaxel *, Napabucasin (BBI608), Gemcitabine, Oxaliplatin, Leucovorin, Irinotecan, Fluorouracil, MM-398 (nanoliposomal irinotecan)	1
22	NCT00666991	Completed	Nanoparticulate paclitaxel	1
23	NCT04233866	Active	Liposomal Irinotecan, Nab-paclitaxel *, Fluorouracilb, Gemcitabine, Leucovorin,	2
24	NCT01300533	Completed	Docetaxel Nanoparticle Targeting Prostate-Specific Membrane Antigen (BIND-014)	1
25	NCT04481204	Active	Nab-paclitaxel *, Cisplatin, Fluorouracil, Gemcitabine, Irinotecan, Leucovorin, Oxaliplatin. Radiation Therapy	2
26	NCT02762981	Completed	Nab-paclitaxel *, Relacorilant	1 2
27	NCT03678883	Active	Nab-paclitaxel *, 9-ING-41(a maleimide-based ATP-competitive small molecule GSK-3β inhibitor), Gemcitabine, Doxorubicin, Lomustine, Carboplatin, Paclitaxel, Irinotecan	2
28	NCT03878524	Active	Nab-paclitaxel * with several antibodies and anticancer drugs.	1
29	NCT03736720	Active	Liposomal Irinotecan, Fluorouracil, Leucovorin	2

* Nab-paclitaxel—a combination of paclitaxel with albumin. It is also known as Abraxane.

## Data Availability

The data presented in this study are available in this article.
